# Mapping QTL hotspots associated with weed competitive traits in backcross population derived from *Oryza sativa* L. and *O. glaberrima* Steud.

**DOI:** 10.1038/s41598-020-78675-7

**Published:** 2020-12-16

**Authors:** Muralidhara Bharamappanavara, Anantha M. Siddaiah, Senguttuvel Ponnuvel, Lokesha Ramappa, Basavaraj Patil, Manoj Appaiah, Sheshu Madhav Maganti, Raman Meenakshi Sundaram, Shashidhar Kadadanamari Shankarappa, Mangal Deep Tuti, Sreedevi Banugu, Brajendra Parmar, Santosha Rathod, Kalyani M. Barbadikar, Suneetha Kota, Lella Venkata Subbarao, Tapan Kumar Mondal, Gireesh Channappa

**Affiliations:** 1 ICAR-Indian Institute of Rice Research, Hyderabad, 500030 India; 2 University of Agricultural Science, Raichur, Karnataka 584104 India; 3 Central Agricultural University, Imphal, 795004 India; 4 ICAR-National Institute for Plant Biotechnology, New Delhi, 110012 India

**Keywords:** Genetics, Molecular biology, Plant sciences

## Abstract

To improve grain yield under direct seeded and aerobic conditions, weed competitive ability of a rice genotype is a key desirable trait. Hence, understanding and dissecting weed competitive associated traits at both morphological and molecular level is important in developing weed competitive varieties. In the present investigation, the QTLs associated with weed competitive traits were identified in BC_1_F_2:3_ population derived from weed competitive accession of *O. glaberrima* (IRGC105187) and *O. sativa* cultivar IR64. The mapping population consisting of 144 segregating lines were phenotyped for 33 weed competitive associated traits under direct seeded condition. Genetic analysis of weed competitive traits carried out in BC_1_F_2:3_ population showed significant variation for the weed competitive traits and predominance of additive gene action. The population was genotyped with 81 genome wide SSR markers and a linkage map covering 1423 cM was constructed. Composite interval mapping analysis identified 72 QTLs linked to 33 weed competitive traits which were spread on the 11 chromosomes. Among 72 QTLs, 59 were found to be major QTLs (> 10% PVE). Of the 59 major QTLs, 38 had favourable allele contributed from the *O. glaberrima* parent. We also observed nine QTL hotspots for weed competitive traits (*qWCA2a*, *qWCA2b*, *qWCA2c*, *qWCA3*, *qWCA5, qWCA7*, *qWCA8*, *qWCA9*, and *qWCA10*) wherein several QTLs co-localised. Our study demonstrates *O. glaberrima* species as potential source for improvement for weed competitive traits in rice and identified QTLs hotspots associated with weed competitive traits.

## Introduction

Rice continuous as major staple food in many parts of world and its consumption is projected to rise 50% by 2050 with a production target of 827 million tonnes^1^. However, irrigated rice ecology, a major supplier of global rice production (94%) is affected due to depleting natural resources, waning water table, mounting labour shortage, increase in production cost and changing climatic condition^2^. These are the major driving forces lead to shift in the cultivation from irrigated rice to direct seeded rice (DSR) as sustainable alternative. DSR sowing is commonly practiced in aerobic and upland rice cultivation and has several advantages such as water saving, reduced labour requirement, increased fertilizer efficiency, reduced destruction of soil properties, early maturity and less methane emission^3^. However, severe weeds infestation^4^ is major threat to the DSR and yield loss of 15% to 100% were reported^5^. Improving weed competitive ability (WCA) through plant breeding efforts is deemed to be safe tool as it offer eco-friendly low-cost weed management coupled with no risk of developing resistant weed biotypes due to herbicides and reduced labour requirement for manual weeding^6–8^. The WCA can be achieved through two components such as weed tolerance and weed suppressive ability^9^. Among two, weed suppressive ability is advocated because suppressing weeds reduces weed seed production and benefits weed management in the future season also^10^. However, strong weed suppression ability does not guarantee higher yields under weed competition due trade off between yield potential and weed competitiveness^11^. Therefore, selection for weed competitiveness along with yield potential should be done simultaneously. There is sufficient genetic variation available in the gene pool even though rice is week competitor^12–21^. However, the progress and genetic gain hampered due to quantitative inheritance, lack of suitable donors, limited knowledge on genetic architecture of WCA^6,22,23^. The WCA of rice cultivars has been attributed to increased plant height, higher tiller number, increased root size, rapid early vigour growth, droopy leaves, high specific leaf area, high leaf area index and allelopathy^11–13,19^. Selection for weed competitiveness can be done directly in the presence of weeds or indirectly under non-competitive conditions for secondary traits related to weed competitiveness^11^.

To dissect the weed competitive traits in rice, QTL mapping has been employed and large number of QTLs for rice WCA traits^2,24^ were identified using biparental segregating populations (F_2_, Backcross inbred line, double haploids, recombinant inbred lines) derived mainly from intrasub-specific^25–28^ and inter-subspecific crosses^29–33^. Recently, association mapping panels^34–37^ and SNP based genotyping by sequencing^38–40^ have been employed. However, no efforts were done to exploit weed competitive QTLs from inherent weed competitive *O. glaberrima* species. African endemic, *O. glaberrima* species has the ability to grow in a wide range of difficult ecosystems and harbours a rich reservoir of genes that allowed it to thrive in the harsh environments^41,42^. Significantly, *O. glaberrima* is known to possess early seedling vigour and weed competitiveness^43–46^ due to faster maturity, high biomass, its wide leaves shade out weeds, weed smothering ability, early seedling vigour, robust root system, its small-diameter roots efficiently extract water and nutrients. Therefore, the African cultivated species will serve as donor species for identification and introgression of weed competitive traits for genetic improvement of Asian cultivated species. However, utilization of *O. glaberrima*, in *O. sativa* breeding is hampered by high sterility in interspecific F_1_ and in early generation of selfed progenies. Repeated backcrossing of interspecific F_1_s with recurrent of *O. sativa* will help in development of fertile progenies. Considering the tremendous scope of utilising the African rice for genetic improvement of Indica rice cultivars, in the present study, weed competitive accession of African rice identified at ICAR-IIRR, Hyderabad (data unpublished) was utilised for development of backcross population and mapping of weed competitive traits.

## Materials and methods

### Plant material and population development

Initially, a total of 31 *O. glaberrima* accessions were evaluated for weed competitive traits under laboratory and direct seeded rice in field conditions for two seasons along with checks (IR64, Sabita, Swarna and BPT5204) (unpublished). Based on findings, one *O. glaberrima* accession (IRGC105187) was found promising for weed competitive traits, namely higher seedling height, biomass and leaf area. Hence, IRGC105187 was used as the donor parent for development of mapping population and IR64, a weed sensitive variety with low seedling vigor was used as the recipient/recurrent parent. In total, 144 BC_1_F_2:3_ families were generated from the cross IR64*1/*O. glaberrima* cross.

### Phenotyping of mapping population for weed competitive traits under direct seeded rice condition

The experiment was laid out in augmented block design with four blocks, wherein, each block consists of 36 BC_1_F_2:3_ families along with five checks. Each family was sown in two rows of two meter length with spacing of 20 × 15 cm. The population was phenotyped for six weed competitive traits such as seedling height (cm), number of tillers, number of leaves, leaf area (cm^2^), shoot fresh weight (g) and shoot dry weight (g). The each observation was made on average of three plants at 15, 30 and 45 days after sowing. The leaf area (cm^2^) was measured using leaf are meter (LI-COR, LI-3100C). Where, fresh leaves of three seedlings were passed through the leaf area meter and average was obtained for each genotype. Similarly, six physiological parameters such as absolute growth rate^36^, relative growth rate^36^, crop growth rate^36^, specific leaf area^47^, leaf area index^47^ and leaf area ratio^47^ were determined at each sampling interval using following formulas.

$${\text{Absolute growth rate }}\left( {{\text{cm day}}^{ - 1} } \right) \, = \frac{{\left( {h2 - h1} \right)}}{{\left( {t2 - t1} \right)}}.$$

where as, “t” is number of days after sowing at which observation was recorded; t_1_ and t_2_ are first and second interval time (e.g., 15 DAS and 30 DAS) of observation respectively. Where, h_1_ and h_2_ are seedling height at t_1_ and t_2_ respectively. The difference between seedling heights of two intervals was divided by difference in days for two sampling intervals and expressed as cm day^-1^.

$${\text{Specific leaf area }}\left( {{\text{cm}}^{2} {\text{g}}^{ - 1} } \right){ } = \frac{{{\text{Leaf area per plant }}\left( {{\text{cm}}^{2} } \right){ }}}{{{\text{Leaf dry weigh per plant }}\left( g \right)}},$$

$${\text{Leaf area index }} = \frac{Leaf area per plant }{{Number of plants m^{ - 2} }},$$

$${\text{Leaf area ratio }}\left( {{\text{cm}}^{2} {\text{g}}^{ - 1} } \right){ } = \frac{{Leaf area per plant \left( {{\text{cm}}^{2} } \right)}}{Total dry weight per plant \left( g \right)},$$

$${\text{Crop growth rate }}\left( {{\text{g m}}^{ - 2} {\text{ day}}^{ - 1} } \right){ } = \frac{{\left( {W2 - W1} \right)}}{{P\left( {t2 - t1} \right)}}.$$

where, w1 and w2 are plant dry weight at times t1 and t2, P = spacing (m^2^).

$${\text{Relative growth rate }}\left( {{\text{g g}}^{ - 1} {\text{ day}}^{ - 1} } \right){ } = \frac{{\left( {loge W2 - loge W1} \right)}}{{\left( {t2 - t1} \right)}},$$

where, w1 and w2 are plant dry weight at times t1 and t2 respectively.

The analysis of variance was carried out in R studio (version 4.0.2) using *augmented RCBD* package^48^ (https://cloud.r-project.org/package=augmentedRCBD) to get augmented ANNOVA and to estimate genotypic coefficient of variability, phenotypic coefficient of variability, broad sense heritability and genetic advance as percent mean. This package also provided histogram plots for each WCA traits. The box plots and correlation plots were plotted using R studio (version 4.0.2) using package ggplot2 (http://ggplot2.tidyverse.org, https://github.com/tidyverse/ggplot2) and ggcorplot (http://www.sthda.com/english/wiki/ggcorrplot) respectively.

### Genotyping of mapping population

Total genomic DNA of 30 days old seedlings was extracted using 2% Cetyl Trimethyl Ammonium Bromide (CTAB) method^49^. The DNA quantity and quality was analyzed by running on 0.8% agarose gel (Biorose agarose) and quantity of DNA in each sample was estimated by comparing the band intensity with known quantity of DNA (Lamda DNA ladder, Takara). The isolated genomic DNA was diluted with 1X TE buffer to get required concentration of DNA (~ 50 ng/μl) in each sample.

A total of 428 SSR markers spanning all over 12 rice chromosomes were used for identification of polymorphic markers between two parents IR64 and *O. glaberrima* (IRGC105187). The SSR marker found polymorphic between parents were used for BC_1_F_2_ population genotyping. The PCR amplification was carried out in Effendorf Vapo.protect PCR cyclers in 96 well plates using following PCR cycling conditions. The 10 μl volume for each reaction containing 3.5 μl of 2X PCR Taq mastermix (ABM with dye), 0.5 μl of each forward and reverse SSR primer (5 pmol), 2 μl of diluted genomic DNA (~ 50 ng/μl) and 3.5 μl of nuclease free water. The PCR cycling involves, initial denaturation (94 °C for 3 min), denaturation (94 °C for 30 s), annealing (50–58 °C for 30 s), extension (72 °C for 40 s) and final extension (72 °C for 5 min) with 35 cycles (step 2, 3 and 4). Finally samples were stored in 4 °C in cyclers. PCR amplified products were resolved in 3–4 per cent agarose and sizes of amplified fragments were determined by comparing with 100 bp ladder (Genei). The documented gels with amplified products were scored visually and allele score “A” was assigned to recurrent parent IR64, allele score “B” was assigned to donor parent *O. glaberrima* and heterozygotes were assigned with allele code of “H”. Whereas, missing alleles were scored as “NA” and non parental alleles as “C”.

### Linkage map construction and QTL analysis

The linkage map was constructed using QTL ICI mapping software v 4.2 (CIMMYT) with MAP function as procedure described by^50^. Recombination frequency of 30 cM was threshold value for grouping, ordering within group was based on K-optimally with 2-optMAP and rippling by recombination frequency with window size of 5. The information regarding marker segregation based on chi-square goodness of fit based also obtained.

The QTL mapping was carried out with Windows QTL cartographer v 2.5 (N.C. state university, Bioinformatics Research Centre).The composite interval mapping methods (CIM) was performed with 1000 permutations and significance level of 0.05 along with the standard model (model 6) of composite interval mapping with forward and backward regression method. The QTLs with threshold of > 2.5 LOD was used as criteria for declaring the QTL. The graphics showing QTL location were obtained from Windows QTL cartographer v 2.5. The standard procedure for QTL nomenclature was outlined by “The committee on Gene Symbolization, Nomenclature and Linkage (CGSNL) of the Rice Genetic Cooperative was followed^51^. Comparison of QTLs with previously reported QTLs were carried out using Q-TARO, Gramene QTL database and research publication (using physical position).

## Results

### Development of interspecific mapping population

The *O. glaberrima* accession, IRGC105187 was crossed to *O. sativa* cv. IR64, a widely adapted mega variety, but poor in weed competitive ability. The interspecific F_1_ developed by crossing IR64 (♀) with *O. glaberrima* (♂ ) showed complete pollen sterility, therefore, the BC_1_F_1_ seeds were generated by backcrossing F_1_ with recurrent parent IR64, wherein, F_1_s served as female parent and IR64 as pollen donor. However, very low seed set during backcross was observed as clipping the florets of the F_1_ plants lead to shattering of florets within 24 h of pollination. The BC_1_F_1_ plants showed partial spikelet fertility (data not shown) and set seeds upon selfing. Each selfed seed obtained from the BC_1_F_1_ plants were raised as individual BC_1_F_2_ plants. Among the BC_1_F_2_ plants the spikelet fertility varied significantly and set seeds upon selfing. The seeds from each BC_1_F_2_ plants were constituted to develop BC_1_F_2:3_ families. In total, 144 BC_1_F_2:3_ families were generated from the cross IR64*1/IRGC105187.

### Phenotyping of BC_1_F_2:3_ mapping population for WCA traits under DSR condition

One hundred forty four BC_1_F_2:3_ families along with five checks grown under direct seeded condition were phenotyped for 12 WCA traits at 15, 30 and 45 DAS. The following abbreviations such as, SH (seedling height), NT (number of tillers), NL (number of leaves), LA (leaf area), SFW (shoot fresh weight), SDW (Shoot dry weight), AGR (absolute growth rate), SLA (specific leaf area), LAI (leaf area index), LAR (leaf area ratio), CGR (crop growth rate), RGR (relative growth rate) with corresponding sampling interval (i.e., SH15, SH30 and SH45) were used hereafter in the manuscript. The augmented analysis of variance (Supplementary Table. S1) revealed significant variability in mapping population for most of the traits except eight WCA traits such as LA15, SLA15, SLA30, LAI15, LAR15, LAR30, LAR45 and CGR15. The checks showed significant difference between each other for all the traits, except for the NT45, NL45 and SLA45. Similarly, mean sum of squares due to treatment vs checks were significant for all traits (SH30, SLA15, SLA30 and SLA45), which indicates that treatments are performing significantly different than checks.

**Figure 1:**
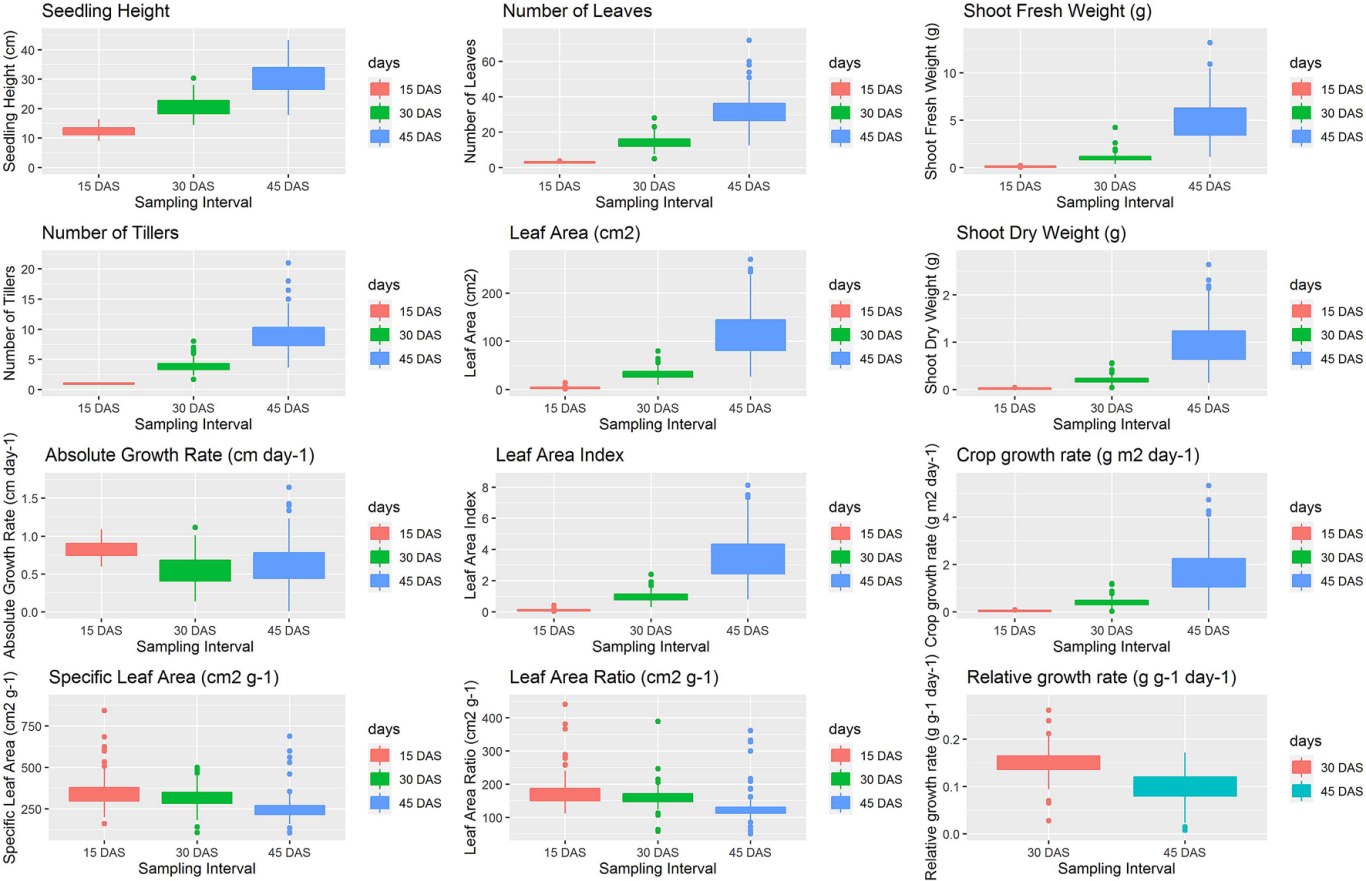
Box plots representing the phenotypic variation for weed competitive ability traits in mapping population.

Phenotypic variation and mean performance of mapping population and parents for WCA traits are presented in Supplementary Table. S2 and graphically represented in box plots (Fig. 1). Although, seedling height in mapping populations was lower at SH15 (12.29 cm) as compared parents, it increased rapidly at SH30 (20.56 cm) and at SH45 (30.30 cm) and recorded higher values than both parents. Number of tiller at NT15 was confined to single tiller per plant. However, average number of tillers were higher than both the parents in mapping population at NT30 (3.99) and NT45 (9.24). Similar findings were observed for number of leaves at NL15, as mapping population including parents had three leaves per plant and average number of leaves in mapping population at NL30 (14.28) and NL45 (32.46) were higher than both the parents. Mean leaf area at LA15 was 3.58 cm^2^, which is lower than both parents. However, significant increase in the leaf area was observed at LA30 and LA45 with mean leaf area of 32.68 cm^2^ and 116.82 cm^2^ respectively. At LA45 average leaf area in the mapping population was higher than the donor parent *O. glaberrima* (105.8 cm^2^). The mean shoot fresh weight (g) of the mapping population at SFW15 was 0.090 g which was lower than both parents. However, significant increase in average shoot fresh weight (g) was observed at SFW30 with 1.04 g and at SFW45 with 5.09 g, which is higher than donor parent *O. glaberrima* (0.91 g and 2.66 g) at both sampling intervals. Similar findings were observed for shoot dry weight (g) at SDW15 with the mapping population recording a mean value of 0.021 g which is lower than both parents. As observed for the other traits, significant increase in average shoot dry weight was observed at SDW30 with 0.210 g and at SDW45 with 0.990 g which is higher than donor parent *O. glaberrima* (0.170 g and 0.497 g) at both sampling intervals. The heritability estimates (Supplementary Table S3) were found high (60.57–98.40%) for all above traits except that it was moderate for SH30, SDW15 (39.66–59.46%) and found low for LA15 (19.45%).

The mean absolute growth rate of the mapping population was observed to decrease from AGR15 (0.81 cm day^−1^) to AGR30 (0.55 cm day^−1^). However, it increased at AGR45 (0.64 cm day^−1^), while IR64 (0.92–0.13 cm day^−1^) and *O. glaberrima* parent (0.88–0.41 cm day^−1^) showed decreasing trends from AGR15-AGR45. The mean specific leaf area of the mapping population was observed to decrease in successive sampling intervals as it reduced from 350.29 cm^2^ g^−1^ at SLA15 to 251.79 cm^2^ g^−1^ at SLA45. Similarly, *O. glaberrima* parent showed decreasing trend from 618.99 cm^2^ g^−1^ at SLA15 to 342.49 cm^2^ g^−1^ at SLA45. The leaf area index found increasing from 0.11 at LAI15 to 3.51 at LAI45. Similarly, *O. glaberrima* parent shown increase in leaf are index from 0.41 at LAI15 to 3.16 at LAI45. The leaf area ratio shown similar trends as of specific leaf area, where mean leaf area ratio was decreased at each sampling interval as it was reduced from 173.27 cm^2^ g^−1^ at LAR15 to 124.98 cm^2^ g^−1^ at LAR45. Similarly, *O. glaberrima* parent shown decreasing trend from 381.82 cm^2^ g^−1^ at LAR15 to 211.26 cm^2^ g^−1^ at LAR45. The mean crop growth rate in mapping population at CGR15 was 0.05 g m^2^ day^−1^ which is lower than both parents. However, crop growth rate was increased at CGR30 with mean of 0.46 g m^2^ day^−1^ and at CGR with mean of 1.73 g m^2^ day^−1^, which is higher than the donor parent *O. glaberrima*. Relative growth rate in mapping population at RGR30 was ranging from 0.07–0.26 g g^−1^ day^−1^ with average of 0.15 g g^−1^ day^−1^. Whereas, at RGR45 it was ranging from 0.01–0.17 g g^−1^ day^−1^ with average of 0.10 g g^−1^ day^−1^. The heritability estimates (Supplementary Table. S3) were found high for AGR15, AGR30, AGR45, LAI30, LAI45, CGR30, CGR45, RGR30 and RGR45 (60.57–98.06%). The traits such as SLA15, SLA30, SLA45, LAR45 and CGR15 had moderate heritability (39.66–59.98%), while heritability was found to be low for LAI15, LAR15 and LAR30 (7.1–20.95%).

All weed competitive traits exhibited positively skewed distribution, except RGR30 and RGR45 which were negatively skewed (Supplementary Fig. S1). Transgressive segregants were observed for all weed competitive traits while the number of families performing better than donor parent increased with later sampling interval for all the traits.

### Correlation among weed competitive ability traits

**Figure 2:**
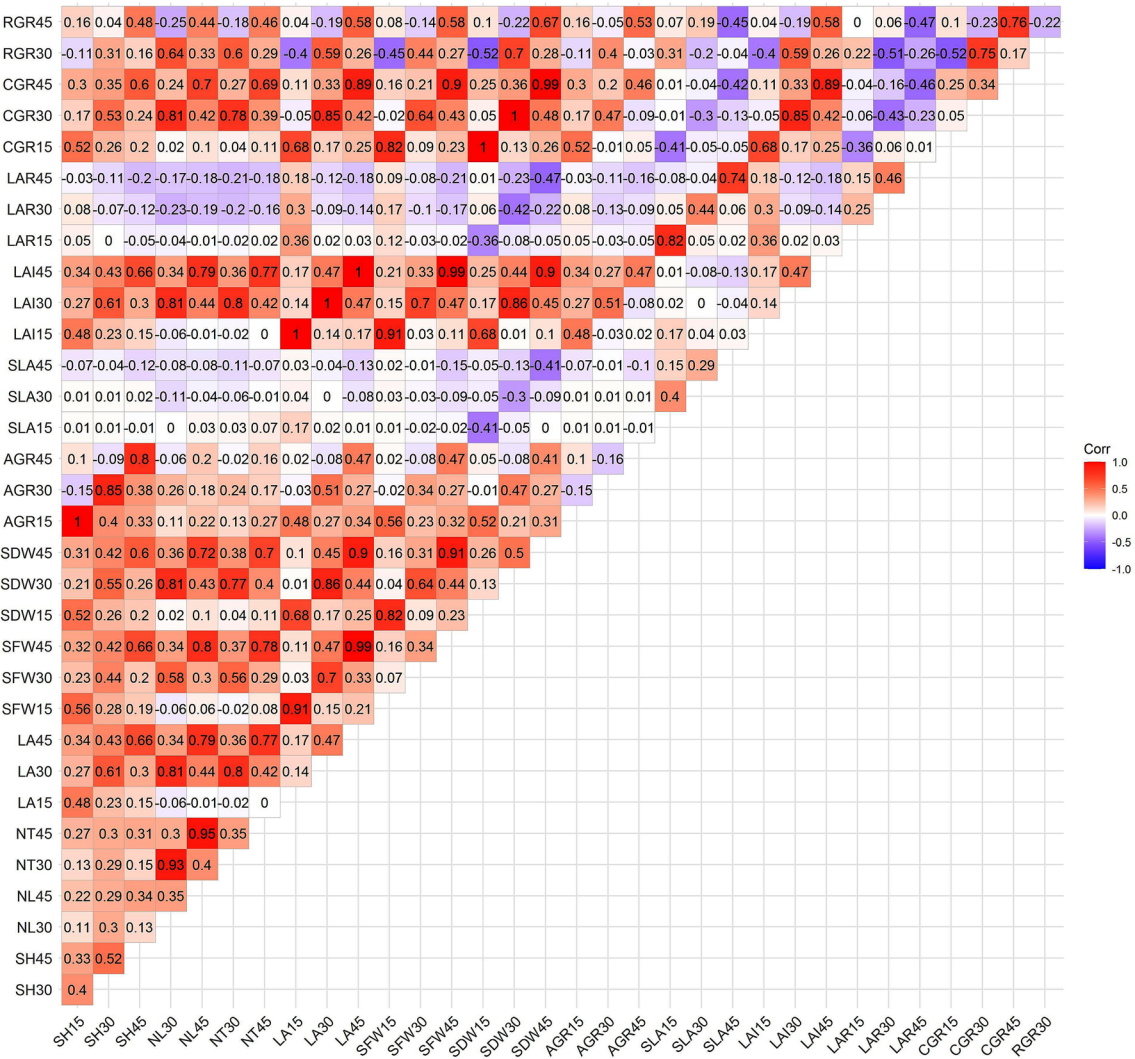
Correlation among weed competitive ability traits in mapping population (Values in the each rectangle indicates correlation coefficients).

The traits such as seedling height, number of tillers, number of leaves, leaf area, shoot fresh weight, shoot dry weight, absolute growth rate, leaf area index and crop growth rate had significant positive association among them at all stages of sampling. However, specific leaf area and leaf area ratio had significant negative association with shoot dry weight, crop growth rate and relative growth rate (Fig. 2).

### Mapping QTLs for weed competitive ability traits

Among 428 SSR markers employed for parental polymorphism between IR64 and IRGC105187, 137 (32%) were found to be polymorphic. Among them 27 markers, which were located at telomere, centromere were excluded. Hence, the mapping population was genotyped with 110 markers. Interestingly, 18 markers were monomorphic (amplifying only IR64 allele) and 11 markers produced non-scorable banding pattern (Supplementary Table S4). Hence, the linkage map was constructed finally with 81 SSR marker data with genome coverage of 1423 cM. The distribution of these 81 polymorphic SSR markers on each chromosome was ranging from three (chromosome 4) to 10 (chromosome 1) with average of seven markers per chromosome. The genome coverage on each chromosome was ranging from 56.39 cM (chromosome 10) to 281.11 cM (chromosome 2) with average genome coverage of 118.60 cM per chromosome. The marker interval ranged from 7.04 cM (chromosome 10) to 35.14 cM (chromosome 2) with average marker interval of 17.76 cM. Significance of chi-square goodness of fit test revealed that, none of the 81 (100%) SSR markers were segregating according to the expected ratio and exhibited segregation distortion with most of the markers showing distortion towards recurrent parent IR64.

The study identified 72 QTLs associated with 12 WCA traits measured at 15, 30 and 45 DAS (Table 1) in the interspecific backcross derived mapping population**.** These QTLs were located on all rice chromosomes except chromosome 1 (Supplementary Table. S5, Supplementary Figure S2). Among the 72 QTLs, 28 QTLs were identified at 15 DAS, 21 QTLs at 30 DAS and 23 QTLs at 45 DAS. There were 59 major QTLs with more than 10% phenotypic variance explained (PVE). Among the 59 major QTLs, 38 QTLs were derived from *O. glaberrima* (Table1). The numbers of QTLs identified for each trait at three sampling intervals are discussed below.

**Table 1:** QTLs identified for weed competitive ability traits in BC_1_F_2:3_ mapping population derived from IR64*1/*O. glaberrima.*

Sl. No	Trait	Chr	QTL Name	Marker Interval	Position (cM)	LOD	PVE (%)	Additive effect	Co-localizations with previous QTLs/genes
1	SH15	12	*qSH15-12.1*	SSR12-7.4- SSR12-4.8	110.30	2.67	23.81	0.87	qCTS12^64^
2	SH30	2	*qSH30-2.1*	RM12434-RM12749	54.20	3.82	6.92	− 0.35	qPH2^65^, qSV2a^38^
3		7	*qSH30-7.1*	RM20866-RM21749	0.00	3.65	12.26	− 1.22	qLTG7^66^, qSL7^67^, qSH12-7.1^31^, qSV7a^38^
4		12	*qSH30-12.1*	RM19-RM27879	12.00	2.57	14.65	− 0.56	qCTS12^64^, qLT12^80^
5	SH45	3	*qSH45-3.1*	RM14735-RM3029	6.00	3.63	6.35	− 4.24	qTAA3-1^25^, RZ313, RG369^69^, qSHL3.1^24^, qSV3b^38^
6	NL30	5	*qNL30-5.1*	RM17816-RM17900	16.00	2.63	9.72	− 1.09	–
7		5	*qNL30-5.2*	RM17900-RM8039	32.20	2.65	25.18	− 1.25	RM13^36^
8	NL45	10	*qNL45-10.1*	RM25796-RM467	24.10	6.55	17.32	− 0.27	Phyllocron^79^
9	NT30	5	*qNT30-5.1*	RM17816-RM17900	16.00	2.84	8.38	− 1.72	–
10		5	*qNT30-5.2*	RM17900-RM8039	34.20	4.74	39.02	− 0.18	qSV5b^38^
11		9	*qNT30-9.1*	RM23901-RM566	10.00	4.06	44.20	0.20	qSV9c^38^
12	NT45	5	*qNT45-5.1*	RM18182-RM5592	51.70	3.24	10.47	− 0.63	nt5.1^78^
13		5	*qNT45-5.2*	RM5592-RM18156	61.40	3.46	11.40	− 1.19	–
14		10	*qNT45-10.1*	RM25796-RM467	24.10	7.35	17.91	− 0.08	qSV10b^38^
15	LA15	2	*qLA15-2.1*	RM12434-RM12749	62.20	3.98	6.52	− 0.58	qLER-2^71^
16		7	*qLA15-7.1*	RM21749-RM22131	20.90	3.86	10.63	− 0.16	QRI7^68^
17		7	*qLA15-7.2*	RM21992-RM5847	28.30	3.38	9.34	− 0.04	QRI7^68^
18	LA30	9	*qLA30-9.1*	RM23901-RM566	10.00	2.54	36.54	0.51	qLA-9^31^
19	LA45	9	*qLA45-9.1*	RM23901-RM566	14.00	2.83	38.89	− 28.08	qLA-9^31^
20	SFW30	4	*qSFW30-4.1*	RM17377-RM3524	44.70	6.57	29.26	− 0.08	qCTS4-3^38^
21		6	*qSFW30-6.1*	RM19819-RM19660	49.00	3.74	14.18	− 0.09	qRS6-1^25^, qSFWd6^70^
22	SFW45	2	*qSFW45-2.1*	RM12434-RM12749	54.20	2.68	4.82	− 0.02	–
23		5	*qSFW45-5.1*	RM18182-RM5592	51.70	2.57	10.84	− 0.59	qLTG5^63^
24	SDW15	2	*qSDW15-2.1*	RM13962-RM1385	163.10	38.86	57.63	0.00	qCSH2 and qSDW2^74^, qSV2c^38^
25		11	*qSDW15-11.1*	RM26558-SSR11.21.1	75.20	2.93	11.96	0.00	qSV11b^38^
26	SDW30	2	*qSDW30-2.1*	RM13155-RM13962	130.80	3.62	12.12	0.02	qCSH2 and qSDW2^74^, qSDWT2.1^33^, RM341^36^, qSV2c^38^
27		9	*qSDW30-9.1*	RM23901-RM566	6.00	5.54	31.85	− 0.01	qSDW9^77^, rdq9^76^, SUB1^75^, qSV9c^38^
28	SDW45	7	*qSDW45-7.1*	RM20866-RM21749	16.00	2.90	27.81	− 0.06	qTN7^80^
29		9	*qSDW45-9.1*	RM23901-RM566	14.00	3.54	47.72	− 0.23	qSV9c^38^
30	AGR15	12	*qAGR15-12.1*	SSR12.7.4- SSR12.4.8	110.30	2.74	24.62	0.06	–
31	AGR30	2	*qAGR30-2.1*	RM12434-RM12749	54.20	3.11	5.77	− 0.01	–
32	AGR45	3	*qAGR45-3.1*	RM14735-RM3029	4.00	3.38	5.21	− 0.27	–
33	SLA15	2	*qSLA15-2.1*	RM13155-RM13962	138.80	5.40	42.71	20.19	–
34		5	*qSLA15-5.1*	RM17900-RM8039	38.20	5.40	10.47	32.68	–
35		5	*qSLA15-5.2*	RM18182-RM5592	51.70	5.66	8.04	− 193.33	–
36		5	*qSLA15-5.3*	RM5592-RM18156	61.40	5.97	4.91	− 198.11	–
37		6	*qSLA15-6.1*	RM19569-RM20181	17.80	7.31	13.24	− 172.93	–
38		7	*qSLA15-7.1*	RM20866-RM21749	18.00	6.33	42.79	85.66	–
39		9	*qSLA15-9.1*	RM23901-RM566	14.00	4.42	45.27	12.16	–
40		10	*qSLA15-10.1*	RM25796-RM467	26.10	7.17	33.82	− 4.30	–
41	SLA30	8	*qSLA30-8.1*	RM38-RM22585	68.10	2.71	3.38	66.32	–
42	SLA45	2	*qSLA45-2.1*	RM13155-RM13962	136.80	14.10	38.73	− 44.01	–
43		2	*qSLA45-2.2*	RM13962-RM1385	151.10	13.34	36.90	− 35.24	–
44		2	*qSLA45-2.3*	RM1385-RM13761	205.40	15.96	11.13	− 162.16	–
45		12	*qSLA45-12.1*	RM27879-RM28067	41.40	16.02	42.42	− 36.61	–
46		12	*qSLA45-12.2*	RM28067-RM28157	51.80	20.05	58.99	− 40.00	–
47	LAI15	7	*qLAI15-7.1*	RM21749-RM22131	20.90	4.69	13.32	0.00	–
48		7	*qLAI15-7.2*	RM21992-RM5847	28.30	4.11	11.77	0.00	–
49	LAI30	9	*qLAI30-9.1*	RM23901-RM566	8.00	2.53	34.40	0.02	–
50	LAI45	9	*qLAI45-9.1*	RM23901-RM566	14.00	2.83	38.90	− 0.84	–
51	LAR15	2	*qLAR15-2.1*	RM13962-RM1385	149.10	7.84	19.60	− 30.07	–
52		3	*qLAR15-3.1*	RM14391-RM15679	57.80	6.68	28.49	− 6.16	–
53		5	*qLAR15-5.1*	RM17900-RM8039	38.20	11.48	17.39	26.38	–
54		5	*qLAR15-5.2*	RM8039-RM18182	43.40	6.71	19.00	− 9.58	–
55		6	*qLAR15-6.1*	RM19569-RM20181	21.80	12.33	52.01	− 27.54	–
56		8	*qLAR15-8.1*	RM433-RM22622	51.10	4.57	13.08	− 8.88	–
57		8	*qLAR15-8.2*	RM38-RM22585	66.10	10.89	39.33	− 12.73	–
58		9	*qLAR15-9.1*	RM23901-RM566	8.00	14.05	53.29	0.64	–
59		10	*qLAR15-10.1*	RM25796-RM467	26.10	13.70	55.08	− 1.39	–
60		11	*qLAR15-11.1*	SSR11-25.5-RM26352	38.00	6.53	19.59	− 37.66	–
61	LAR30	8	*qLAR30-8.1*	RM38-RM22585	66.10	5.39	29.13	21.38	–
62		9	*qLAR30-9.1*	RM23901-RM566	6.00	5.73	29.71	6.08	–
63		12	*qLAR30-12.1*	RM19-RM27879	24.00	6.49	15.27	43.83	–
64	LAR45	12	*qLAR45-12.1*	RM27879-RM28067	43.40	19.23	31.60	− 10.44	–
65		12	*qLAR45-12.2*	RM28067-RM28157	51.80	24.55	30.60	− 10.56	–
66	CGR15	11	*qCGR15-11.1*	RM26558-SSR11-21.1	75.20	5.23	18.26	− 0.03	–
67	CGR30	2	*qCGR30-2.1*	RM13155-RM13962	130.80	3.45	12.82	0.04	–
68		9	*qCGR30-9.1*	RM23901-RM566	6.00	5.26	31.34	− 0.02	–
69	CGR45	2	*qCGR45-2.1*	RM13962-RM1385	159.10	2.70	47.50	− 0.08	–
70		5	*qCGR45-5.1*	RM17900-RM8039	36.20	3.44	36.27	0.54	–
71		9	*qCGR45-9.1*	RM23901-RM566	14.00	3.66	43.80	− 0.24	–
72	RGR45	3	*qRGR45-3.1*	RM14735-RM3029	0.00	2.84	3.20	− 0.02	–

*PVE (%)* Phenotypic variance explained.

A total of five QTLs were identified for seedling height across three sampling stages namely, *qSH15-12.1* (23.81%), *qSH30-7.1* (12.26%), *qSH30-12.1* (14.65%), *qSH30-2.1* (6.92%) and *qSH45-3.1* (6.35%). Six QTLs were identified for number of tillers at NT30 and NT45 namely *qNT30-5.1* (8.38%), *qNT30-5.2* (39.02%), *qNT30-9.1* (44.20%), *qNT45-5.1* (10.47%), *qNT45-5.2* (11.40%) and *qNT45-10.1* (17.91%). Number of leaves had three QTLs at NL30 and NL45 such as *qNL30-5.1* (9.72%), *qNL30-5.2* (25.18%) and QTL *qNL45-10.1* (17.32%).The leaf area had five QTLs across three sampling stages, namely *qLA15-7.1* (10.63%), *qLA15-2.1* (6.52%) and *qLA15-7.2* (9.34%), *qLA30-9.1* (36.54%) and *qLA45-9.1* (38.89%). Shoot fresh weight had two QTLs each at SFW30 and SFW45 namely *qSFW30-4.1* (29.26%), *qSFW30-6.1* (14.18%), QTL *qSFW45-2.1* (4.82%) and *qSFW45-5.1* (10.84%). There were six QTLs for total for shoot dry weight across three sampling stages, viz., *qSDW15-2.1* (57.63%), *qSDW15-11.1* (11.96%), *qSDW30-2.1* (12.12%), *qSDW30-9.1* (31.85%), *qSDW45-7.*1 (27.81%) and *qSDW45-9.*1 (47.72%).

The trait absolute growth rate had one QTL each at three sampling intervals, viz., *qAGR15-12.1* (24.62%), *qAGR30-2.1* (5.77%) and *qAGR45-3.1* (5.21%). Relatively large number of (14) QTLs were identified for specific leaf area at three sampling stages. QTLs such as *qSLA15-2.1, qSLA15-5.1, qSLA15-6.1, qSLA15-7.1, qSLA15-9.1 qSLA15-10.1* had phenotypic variance ranging from 10.47–45.27%. At SLA30, one minor QTL *qSLA30-8.1* (3.38%) was identified. Whereas at SLA45, five major QTLs were found (*qSLA45-2.1, qSLA45-2.2, qSLA45-2.3, qSLA45-12.1* and *qSLA45-12.2*) with phenotypic variance of 11.13–58.99%. Four major QTLs namely *qLAI15-7.1* (13.32%) *qLAI15-7.1* (11.77%), *qLAI30-9.1* (34.40%) and *qLAI45-9.1* (38.90%) were identified for leaf area index. The trait leaf area ratio had 15 QTLs, wherein 10 QTLs were detected at LAR15, three QTLs at LAR30 and two QTLs at LAR45. The QTLs *qLAR15-2.1, qLAR15-3.1, qLAR15-5.1, qLAR15-5.2, qLAR15-6.1, qLAR15-8.1, qLAR15-8.2, qLAR15-9.1, qLAR15-10.1* and *qLAR15-11.1* had phenotypic variance of 13.08–52.01%. The QTLs at LAR30 (*qLAR30-8.1, qLAR30-9.1* and *qLAR30-12.1*) had phenotypic variance of 15.27–29.71%. Whereas, at LAR45 two major QTLs, *qLAR45-12.1* (31.60%) and *qLAR45-12.2* (30.60%) were identified. Across three sampling intervals, six QTLs were identified for crop growth rate namely *qCGR15-11.1* (18.26%), *qCGR30-2.1* (12.82%), *qCGR30-9.1* (31.34%), *qCGR45-2.1* (47.50%), *qCGR45-5.1* (36.27%) and *qCGR45-9.1* (43.80%). The relative growth rate had one minor QTL (*qRGR45-3.1*) on chromosome three with phenotypic variance of 3.20%.

### QTL hotspots for weed competitive ability traits

Interestingly, the QTL analysis identified nine genomic regions as QTL hotspot, wherein several QTLs associated with weed competitive traits were found to be co-localized. Among the nine QTL hotpots, three hotspots were localised on chromosome 2 while one each QTL hotspot on chromosome 3, 5, 7, 8, 9, 10 (Table 2). Three QTL hotspots of chromosome 2 were located between RM12434-RM12749, RM13155-RM13962 and RM13962-RM13585 harbouring 4 (*qSH30-2.1, qFW45-2.1, qAGR30-2.1* and *qLA15-2.1*), 4 (*qSDW30-2.1, qSLA15-2.1, qSLA45-2.1* and *qCGR30-2.1*) and 4 (*qSDW15-2.1, qSLA45-2.2, qLAR15-2.1* and *qCGR45-2.1*) QTLs respectively. The QTL hotspot on chromosome 9 harboured 12 QTLs (*qSDW30-9.1, qLAR30-9.1, qCGR30-9.1, qLAI30-9.1, qLAR15-9.1, qNT30-9.1, qLA30-9.1, qLA45-9.1, qSDW45-9.1, qSLA15-9.1, qLAI45-9.1* and *qCGR45-9.1*) was located between RM239001-RM566. There were eight QTLs (*qNL30-5.2, qNT30-5.2, qSLA15-5.1, qLAR15-5.1*, *qCGR45-5.1, qNT45-5.1, qFW45-5.1* and *qSLA15-5.2*) co-localized on chromosome 5 between RM17900-RM5592. Similarly, chromosome 10 had for four co-localized QTLs (*qNL45-10.1, qNT45-10.1, qSLA15-10.1* and *qLAR15-10.1*) between RM25796-RM467. In case of QTL hotspot on chromosome 3 (RM14735-RM3029), chromosome 7 (RM20866-RM21749) and chromosome 8 (RM38-RM22585), the number of co-localized QTLs were 3 (*qSH45-3.1, qAGR45-3.1* and *qRGR45-3.1*), 3 (*qSH30-7.1, qSDW45-7.1* and q*SLA15-7.1*), and 3 (*qSLA30-8.1, qLAR15-8.2* and *qLAR30-8.1*) QTLs respectively.

**Table 2:** QTL hotspots identified for weed competitive ability traits.

Sl. No	Chr	QTL hotspot	Genomic region	Position (cM)	Physical size (Mb)	No. of QTLs	Co-localized QTLs
1	2	*qWCA2a*	RM12434-RM12749	61.59–115.44	5.03	4	*qSH30-2.1, qFW45-2.1, qAGR30-2.1* and *qLA15-2.1*
2	2	*qWCA2b*	RM13155-RM13962	153.75–186.77	16.0	4	*qSDW30-2.1, qSLA15-2.1, qSLA45-2.1* and *qCGR30-2.1*
3	2	*qWCA2c*	RM13962-RM13585	186.77–269.77	4.70	4	*qSDW15-2.1, qSLA45-2.2, qLAR15-2.1* and *qCGR45-2.1*
4	3	*qWCA3*	RM14735-RM3029	0.00–16.95	2.65	3	*qSH45-3.1, qAGR45-3.1* and *qRGR45-3.1*
5	5	*qWCA5*	RM17900-RM5592	21.37–79.86	19.19	8	*qNL30-5.2, qNT30-5.2, qSLA15-5.1, qLAR15-5.1*, *qCGR45-5.1, qNT45-5.1, qFW45-5.1* and *qSLA15-5.2*
6	7	*qWCA7*	RM20866-RM21749	0.00–25.14	19.32	3	*qSH30-7.1, qSDW45-7.1* and q*SLA15-7.1*
7	8	*qWCA8*	RM38-RM22585	84.98–90.53	3.97	3	*qSLA30-8.1, qLAR15-8.2* and *qLAR30-8.1*
8	9	*qWCA9*	RM23901-RM566	4.12–69.84	7.71	12	*qSDW30-9.1, qLAR30-9.1, qCGR30-9.1, qLAI30-9.1, qLAR15-9.1, qNT30-9.1, qLA30-9.1, qLA45-9.1, qSDW45-9.1, qSLA15-9.1, qLAI45-9.1* and *qCGR45-9.1*
9	10	*qWCA10*	RM25796-RM467	26.10–30.82	7.61	4	*qNL45-10.1, qNT45-10.1, qSLA15-10.1* and *qLAR15-10.1,*

## Discussion

Even though scarcity of labour and water is forcing shifting of rice cultivation from irrigated to direct seeded, it is besotted with major production constraints like weed infestation. Hence, development of weed competitive cultivars through plant breeding has become imperative for sustainable production under direct seeded and aerobic rice conditions. However, weed competitive ability is a quantitative trait, determined by interaction of associated traits such as plant height, tiller number, leaf canopy traits and root traits. Hence, insight into the genetic and molecular mechanisms of weed competitive ability will help in rapidly developing weed competitive cultivars. Therefore, the present study was designed and carried out to evaluate weed competitive ability of a mapping population derived from *O. sativa* × *O. glaberrima* cross and to dissect their association with chromosomal region using QTLs identification approach.

### Performance of mapping population for weed competitive ability traits

Based on mean performance, it was observed that, most of the lines in mapping population had lower seedling height than both parents at SH15. However, mapping population displayed rapid increase in the seedling height at SH30 and SH45 as compared to the parents. It was evidenced by higher absolute growth rate of mapping population as compared to the recurrent parent (i.e. IR64) at AGR30 and both the parents at AGR45. Vigorous growth of *O. glaberrima* accessions after initial establishment has been well documented in earlier studies^12,43,52^. Recently, a study^53^ reported significant variability for plant height (8 DAS-28DAS) in introgression lines derived from *O. glaberrima*. The mean number of tillers and number of leaves were found higher than both parents at 30 DAS and 45 DAS. The higher number of tillers and leaves in the mapping population expected to produce large canopy and increase in the ground coverage at early stage of crop development. However, a study^52^ reported lower number of tillers in the mapping population as compared to *O. glaberrima* parent in inter-specific cross derived progenies. The shoot fresh weight and dry weight was on lower side than that both the parents at SFW15 and SDW15. However, significant improvement over parents was observed at 30 and 45 DAS sampling as mapping population produced more biomass at exponential rate. The increased biomass accumulation in introgression lines can be attributed to rapid increase in the seedling height, tiller number, number of leaves at 30 and 45 DAS. The higher biomass indicates ability of genotype to produce more plant organs for given amount of assimilates. However, a study^52^ observed intermediate dry weight values for *O. glaberrima* derived inter-specific progenies. In support to our observations in the this study, a recent study^53^ reported higher dry biomass accumulation in introgression lines of *O. glaberrima* at 28 DAS. Rapid biomass accumulation in mapping population was evidenced by higher means of crop growth rate and relative growth rate in mapping population at 30 DAS and 45 DAS. The leaf area at 15 DAS did not show any significant variability in the mapping population and none of introgression lines were found better than the *O. glaberrima* parent. However, range of phenotypic variability significantly widened at later stages and mapping population had higher mean leaf area than IR64 at 30 DAS and both parents at 45 DAS. These results indicate introgression lines/progenies have the ability to put up more rapid leaf area. In support to our findings, a study^43^ suggested the rapid initial growth of *O. glaberrima* interspecific progenies was associated with faster leaf growth. Another study^54^ also reported large variability for leaf area in introgression lines. Our findings showed similar trends with respect to leaf area index. Specific leaf area at 15 DAS showed that, most of the introgression lines had higher specific leaf area than the recurrent parent IR64 but lesser than the *O. glaberrima* parent. However, at later sampling stages (30 and 45 DAS), mean specific leaf area of mapping population shown decreasing trends. These results indicate leaves will get thicker as growth progresses and found to be having intermediary values of both parents. As opined by Dingkhun^45^, cultivars that have large specific leaf area during early developmental stages (more ground coverage) and small specific leaf area (for yield benefit) during advance stage is desirable for weed competitiveness. Several earlier studies^43,44^ have reported *O. glaberrima* interspecific progenies had intermediate specific leaf area during early growth stages, followed by a decrease as that of *O. sativa* parent. Similar results were also reported^52,54^ in *O. glaberrima* interspecific progenies. In our study, the mapping population had lower leaf area ratio than both the parents at all sampling stages. It indicates mapping population produced less leaf area per unit dry weight and same dry matter might be diverted to development of seedling height, tillers and leaves. There is lack of reports on leaf area ratio in *O. glaberrima* and their progenies.

### Interspecific linkage map

The result found that, 137 out of 428 (32%) SSR markers were polymorphic. The polymorphism% in the present study was found to be low when compared to other studies involving inter-specific cross between *O. sativa* × *O. glaberrima* parents. For instance a study^55^ reported 100 out of 140 (71.40%) SSRs were polymorphic, while other^56,57^ reported higher an even higher 79.3% (130 out of 164) and 77.9% (109 out of 140 microsatellites) polymorphism, respectively. However, there are studies^58,59^, which reported lower level of polymorphism (27–38% and ~ 40% respectively). Another study^60^, reported polymorphism % ranging from 23.87 to 50.66%. Based on these reports, there is a large variation for polymorphism % and results are confined to specific particular studies and they cannot be generalized. The factors such as, extent of genetic diversity between the parents, gene pool they belong to and distribution of chosen markers on chromosome affects results of polymorphism.

In our study, linkage map with length of 1423 cM was constructed using 81 polymorphic SSR markers. The length of linkage map and marker interval varies with number of markers used, recombination between the markers and population type. Hence, there is no common agreement between linkage maps of different studies which used *O. glaberrima* as donor parent. The first interspecific *O. sativa* × *O. glaberrima* microsatellite based genetic linkage map was of 1923 cM (129 markers)^61^. Later studies reported linkage map of 1050 cM (100 SSR markers) with 10.5 cM marker interval in O. sativa*O. glaberrima population (Caiapo × IRGC 103,544)^55^. Similarly^56,57^ reported linkage map of 1725 cM (130 SSR markers) and 1162 cM (60 SSR markers) length respectively in interspecific rice populations derived from *O. sativa* × *O. glaberrima* (WAB56-104 × CG14 and IR64 × TOG5681)*.* Other studies^59,60^ reported physical map length of 371 Mb (86 SSR and 87 STS markers) and linkage map length of 2183.13 cM (114 microsatellites) in interspecific populations respectively. Very recently, a study^62^ reported linkage map with length of 2426.17 cM using 652 SNP markers.

### *O. glaberrima* as source of weed competitiveness ability traits

In the present study, 59 major QTLs were detected with phenotypic variance of 10.47–58.99%. Among the 59 major QTLs, 38 QTLs were derived from *O. glaberrima*. These 38 major QTLs from *O. glaberrima* were identified for weed competitive traits such as seedling height, number of tillers, number of leaves, leaf area, shoot fresh weight, shoot dry weight, specific leaf area, leaf area index, leaf area ratio and crop growth rate. Whereas, QTLs from *O. glaberrima* for traits such as absolute growth rate and relative growth rate were observed to be minor. The QTLs such as *qSH30-7.1* (qLTG7^66^, qSL7^67^, qSH12-7.1^31^, qSV7a^38^), *qSH45-3.1* (qTAA3-1^25^, RZ313, RG369^69^, qSHL3.1^24^, qSV3b^38^), *qNT30-5.2*(qSV5b^38^), *qNT45-10.1* (qSV10b^38^), *qLA30-9.1* & *qLA45-9.1* (qLA-9^31^), *qSFW30-6.1* (qRS6-1^25^, qSFWd6^70^) and *qSDW30-9.1* & *qSDW45-9.1* (qSDW9^77^, rdq9^76^, SUB1^75^, qSV9c^38^) were had beneficial alleles from *O. glaberrima* with major QTL effects. Hence, these QTLs can be employed in marker-assisted selection for developing weed competitive cultivars. These results indicates, *O. glaberrima* species inherits genes/allele essential for development of weed competitive traits and could be used as potential donor in breeding for weed competitiveness. The QTLs identified in present study will pave for development of weed competitive rice genotypes suitable for DSR.

### Dynamic expression of weed competitive QTLs

In rice, several studies have reported QTL associated with WCA traits present on 12 chromosomes^24–40^. Each study used different determinants to assess and identify genomic regions associated with WCA viz*.,* shoot length, shoot weight, coleoptile length, shoot dry weight, leaf area and specific leaf area. Most of the QTLs identified for WCA traits have been carried out under controlled conditions using petri dishes, slant plates, paper rolls, hydroponics, sand and hydroponics and soil filled pipes^2^, while the major QTLs associated with WCA traits were identified through a thorough field screening experiment. It was also observed that, traits such as seedling/plant height and shoot/total dry weight were used as major determinants of WCA in most the studies and large number of QTLs were reported for these two traits. The traits such as fresh biomass, number of leaves and leaf area were also given relative importance. However, trait such as number of tillers and physiological parameters such as specific leaf area, growth rate estimates were sparsely used and relatively less number of QTLs were reported.

In our study, relatively equal number of QTLs was identified at each sampling stage. Among the 72 QTLs, 28 QTLs were identified at 15 DAS, 21 QTLs at 30 DAS and 23 QTLs at 45 DAS. It was also observed that, QTLs shown stage specific or dynamic expression as none of QTL region commonly found across three sampling stages. However, QTL region on chromosome 9 (RM23901-RM566) found common at two sampling intervals for several traits such as LA30, LA45, SDW30, SDW45, LAI30, LAI45, LAR15, LAR30, CGR30 and CGR45. Similarly, QTL region on chromosome 2 (RM13155-RM13962) found common SLA15 and SLA45. These stable QTLs can be further analysed and characterized for future studies. Similar to our findings, stage specific or dynamic expression of WCA QTLs has been well documented in previous studies. Recently a study^38^ reported that, 33.3% (plant height), 10.7% (tiller number) and 3.4% (above ground dry weight) of the total QTLs were detected in all three sampling stages. Our study indicates that, QTL identified are specific to either 15 DAS or 30 or 45 DAS and we could not find common QTLs across all three stages. Therefore, it can be concluded that QTLs expression is highly dynamic and stage specific. The consistent QTLs and stage specific QTLs associated with weed competitive in our study plays significant role in understanding the genetic architecture of weed competitive traits.

### Comparison of QTLs identified in the present study with those from previous reports

The QTLs identified in the present study were compared with previously reported QTLs (using QTARO, Gramene QTL database and research reports with physical positions). The QTLs identified for cold or low temperature tolerance/submergence tolerance/drought tolerance in the previous studies were taken into consideration as they are directly correlated with vigour^24,31,33,36,38,63–80^. Based on these findings, QTLs identified in the present study for traits such as seedling height, number of leaves, number of tillers, leaf area, shoot fresh weight, shoot dry weight at all sampling co-localized with those identified in previous studies except *qNL30-5.1, qNT30-5.1 and qSFW45-2.1*. These three minor QTLs were derived from *O. glaberrima* parent and found to be novel. As most of the identified QTLs were co-localized with previous reports, which are based on different mapping population and diverse environments, the QTLs identified in the present study can be utilized in developing WCA varieties with greater confidence. However, QTLs for the traits such as absolute growth rate, specific leaf area, leaf area index, leaf area ratio crop growth rate and relative growth rate have been sparsely studied so far and only few QTLs have been reported with respect to WCA in rice.

### QTL hotspots for weed competitive ability traits

The QTL “hotspots” for WCA and related traits were reported by many studies^23,24,29,30,36,67,70^ on different chromosomes for various co-localized traits. The possible reasons for co-localization may be due to linkage or pleiotropy. Similarly, significant correlations among co-localized traits provide possible explanation for QTL “hotspots”. The study^25^ also pointed out that, co-localization may have occurred by chance, whenever large numbers of QTLs were detected. In the present study, QTL hotspot *qWCA9* (7.71 Mb) harboured 12 major QTLs with average phenotypic variance of 39.65%. Whereas, QTL hotspot *qWCA5* (19.19 Mb) had eight co-localized QTLs with average phenotypic variance of 18.66%. The QTL hotspots on chromosome 2, *qWCA2a* (5.03 Mb), *qWCA2b* (16.0 Mb), and *qWCA2c* (4.70 Mb) found to harbour four QTLs each with average phenotypic variance of 6.0%, 26.59% and 40.40%. However, all the QTLs in *qWCA2a* and *qWCA2c* had positive alleles from *O. glaberrima* and in *qWCA2b*, recurrent parent IR64 contributed positive alleles. Similarly, the QTL hotspot *qWCA10* (7.61 Mb) co-localized with four major QTLs contributed from *O. glaberrima* with average phenotypic variance of 31.03%. The QTL hotspot *qWCA3* (2.65 Mb) and *qWCA5* (13.17 Mb) had three QTLs each with average phenotypic variance of 4.92% and 9.78% respectively with positive alleles contribution from *O. glaberrima*. While, QTL hotspots *qWCA7* (19.32 Mb) and *qWCA8* (3.97 Mb) had average phenotypic variance of 27.62% and 23.94% respectively with positive alleles contribution from both parents. These results indicate both parents associated with positive alleles of WCA associated traits and presence wide range of molecular and phenotypic diversity for WCA associated traits among mapping population^23^. These genomic regions need to be further characterized and used as potential target in marker assisted breeding for improvement of rice varieties for WCA. However, QTL hotspots identified in multiple environments could be ideal for understanding its regulatory role and further use in molecular breeding.

### Segregation distortion in *O. sativa* × *O. glaberrima* mapping population

Segregation distortion (deviation of observation of marker ratio from the expected ratio) is the strong evolutionary force, commonly encountered in mapping populations derived from diverse genotypes^81,82^. In QTL mapping, the segregation distortion known to affect precision of QTL mapping by altering the genetic distance between the markers and the order of the markers on the linkage group. However, Zhang et al.,^83^ concluded that, in general, segregation distortion will not produce more false QTL, nor will it have significant impact on the estimation of QTL position and effect. As Zhang et al.^83^ and Xu^84^ suggested, dense linkage map with large-size mapping population would minimize power loss even in the presence of segregation distortion. In the present study, mapping population showed presence of segregation distortion for all the markers used. Presence of hybrid sterility genes and gametophyte competition gene^55,59,61,85^ in *O. sativa* × *O. glaberrima* interspecific population were found to cause segregation distortion. A recent study^85^ reported 10 hybrid sterility loci (*S1*-glab, *S19*-glab, *S20*-glab, *S37*-glab, *S38*-glab, *S39*-glab) as gamete eliminator or pollen killer between *O. sativa* × *O. glaberrima* interspecific population. We also observed high amount of spikelet sterility in the backcross progenies. Similar to our findings, several studied in have reported presence of sterility and segregation distortion in *O. sativa* × *O. glaberrima* interspecific populations^55,59,61,62^. Very recently, Neelam et al.^62^, reported segregation distortion in SNP genotyping while mapping QTL for bacterial leaf blight from *O. glaberrima* derived population. As Xu and Hu^86^ opined, for a long period of time distorted markers were simply discarded from QTL mapping for the reason of precaution and they also found that if distorted markers handled properly, they can be beneficial to QTL mapping with no detrimental effects. Previous studies used statistical package Proc QTL^86^ (SAS) and MapDisto 2.0^55^ for handling of segregation distortion in F_2_ and BC_1_F_1_, RIL and DH population only. However, there is a need develop suitable package for BC_1_F_2_ population. The QTLs identified in the present study can be considered as least affected by segregation distortion as most of the QTLs identified were co-localized with previously identified QTLs. In support of our observation, Xian-Liang^81^ also opined that, effect of segregation distortion is minimal with the use of co-dominant markers like SSRs and also in backcross population.

## Conclusions

Shift towards DSR cultivation has been gaining importance in recent years, which offer considerable saving of water and labour resources. However, it inherited the threat of weed infestation. Hence, breeding for weed competitive rice cultivars is need of the hour to tackle problem of weed infestation under DSR. The African endemic rice species *O. glaberrima* has the weed competitive ability due to their early vigorous growth and high specific leaf area. However, there are no systemic studies till date to identify the genomic regions (QTLs/genes) associated with weed competitiveness in *O. glaberrima.* The present study is, first of its kind, in which inter-specific mapping population developed from IR64 × *O. glaberrima* (BC_1_F_2:3_) was employed to dissect QTLs for WCA traits. Phenotyping of mapping population revealed significant variability and presence of transgressive segregants for all the traits under study. Based on SSR genotyping linkage map of 1423 cM was constructed using 81 polymorphic markers which showed segregation distortion. The composite interval mapping identified 72 QTLs for 33 WCA traits which are stage specific. Of the 72 QTLs, 59 found to be major QTLs with more than 10% PVE. Among the 59 major QTLs, 38 QTLs were derived from *O. glaberrima*. These results indicate superiority of *O. glaberrima* in contributing favourable alleles for WCA traits. The study identified nine QTLs hotspot in the genome, wherein at least three QTLs were co-localized. Further, there is need for validation of putative QTLs, identification of candidate genes in QTL hot spots, fine mapping of major QTLs and development of functional markers in exploiting *O. glaberrima* for WCA traits.

## Supplementary Information

Supplementary Information
