# Supplementary material for: Mapping QTL hotspots associated with weed competitive traits in backcross population derived from Oryza sativa L. and O. glaberrima Steud
Source: Sci Rep. 2020 Dec 16;10:22103. doi: 10.1038/s41598-020-78675-7 (PMC7744529; doi:10.1038/s41598-020-78675-7)
Supplement: Supplementary file 1 — Supplementary Information [file 41598_2020_78675_MOESM1_ESM.docx]

**Supplementary information**

**Mapping QTL hotspots associated with weed competitive traits in backcross population derived from *Oryza sativa* L. and *O. glaberrima* Steud.**

Muralidhara Bharamappanavara^2#^, Anantha Siddaiah^1^, Senguttuvel Ponnuvel^1^, Lokesha Ramappa^2^, Basavaraj Patil^1^, Manoj Appaiah^1^, Sheshu Madhav Maganti^1^, Raman Meenakshi Sundaram^1^, Shashidhara Shankarappa^3^, Mangal Deep Tuti^1^, Sreedevi Banugu^1^, Brajendra Parmar^1^, Santosha Rathod^1^, Kalyani Kulakarni^1^, Suneetha Kota^1^, Lella Venkata Subbarao ^1^, Tapan Kumar Mondal^4^ and Gireesh Channappa^1*#^

^1^ ICAR-Indian Institute of Rice Research, Hyderabad, India, 500030

^2^ University of Agricultural Science, Raichur, Karnataka, India, 584104

^3^ Central Agricultural University, Imphal 795004.

^4^ ICAR-National Institute for Plant Biotechnology, 110012.

# Authors contributed equally

^*^Corresponding author:giri09@gmail.com

**Supplementary Table S1: Analysis of variance for weed competitive traits**

| **Source** | **DF** | **SH15** | **SH30** | **SH45** | **NT30** | **NT45** | **NL30** | **NL45** | **LA15** |
| --- | --- | --- | --- | --- | --- | --- | --- | --- | --- |
| Block (ignoring Treatments) | 3 | 16.31 ** | 12.53 * | 6.83 ns | 1.99 ** | 55.09 ** | 26.48 ** | 467.39 ** | 6.39 ** |
| Treatment (eliminating Blocks) | 148 | 3.25 * | 12.39 ** | 30.19 ** | 1.02 ** | 7.13 ** | 15.72 ** | 91.51 ** | 3.34 ** |
| Treatment: Check | 4 | 34.53 ** | 165.4 ** | 172.4 ** | 0.78 * | 4.62 ns | 7.81 * | 47.22 ns | 49.67 ** |
| Treatment: Test and Test vs. Check | 144 | 2.38 * | 8.14 * | 26.23 ** | 1.03 ** | 7.2 ** | 15.94 ** | 92.74 ** | 2.05 ** |
| Treatment (ignoring Blocks) | 148 | 3.57 ** | 12.63 ** | 29.95 ** | 1.06 ** | 8.23 ** | 16.18 ** | 100.87 ** | 3.29 ** |
| Treatment: Test vs. Check | 1 | 23.98 ** | 0.02 ns | 262.81 ** | 27.19 ** | 258.65 ** | 603.82 ** | 3859.29 ** | 205.23 ** |
| Treatment: Test | 143 | 2.56 * | 8.45 * | 24.34 ** | 0.88 ** | 6.58 ** | 12.3 ** | 76.09 * | 0.58 ns |
| Block (eliminating Treatments) | 3 | 0.61 ns | 0.62 ns | 18.52 * | 0.0042 ns | 0.74 ns | 3.66 ns | 5.68 ns | 8.73 ** |
| Residuals | 12 | 1.01 | 3.42 | 3.25 | 0.22 | 1.85 | 2.26 | 22.65 | 0.47 |
| CV (%)@0.05 |  | 8.08 | 9 | 6.05 | 12.2 | 15.5 | 11.08 | 15.52 | 17.13 |

| **Source** | **DF** | **LA30** | **LA45** | **SFW15** | **SFW30** | **SFW45** | **SDW15** | **SDW30** | **SDW45** |
| --- | --- | --- | --- | --- | --- | --- | --- | --- | --- |
| Block (ignoring Treatments) | 3 | 390.7 ** | 11692.24 ** | 0.0029 ** | 0.13 * | 15.43 ** | 0.00025 ** | 0.01 ** | 1.17 ** |
| Treatment (eliminating Blocks) | 148 | 126.03 ** | 2369.37 ** | 0.00091 ** | 0.21 ** | 4.91 * | 4.5e-05 ns | 0.01 ** | 0.23 ** |
| Treatment: Check | 4 | 435.44 ** | 5454.39 ** | 0.01 ** | 0.21 ** | 10.85 ** | 0.00041 ** | 0.02 ** | 0.24 ** |
| Treatment: Test and Test vs. Check | 144 | 117.44 ** | 2283.67 ** | 0.00054 ** | 0.21 ** | 4.75 * | 3.5e-05 ns | 0.01 ** | 0.23 ** |
| Treatment (ignoring Blocks) | 148 | 133.92 ** | 2604.04 ** | 0.00091 ** | 0.21 ** | 5.04 * | 4.5e-05 ns | 0.01 ** | 0.25 ** |
| Treatment: Test vs. Check | 1 | 1605.19 ** | 49016.82 ** | 0.03 ** | 3.79 ** | 75.52 ** | 0.00028 ** | 0.18 ** | 6.96 ** |
| Treatment: Test | 143 | 115.2 ** | 2199.75 ** | 0.00031 * | 0.19 ** | 4.39 * | 3.3e-05 ns | 0.01 ** | 0.21 ** |
| Block (eliminating Treatments) | 3 | 1.45 ns | 114.89 ns | 0.003 ** | 0.01 ns | 9 ** | 0.00023 ** | 0.00025 ns | 0.03 ** |
| Residuals | 12 | 8.43 | 126.18 | 9.60E-05 | 0.03 | 1.49 | 2.00E-05 | 0.00063 | 0.0033 |
| CV (%)@0.05 |  | 9.21 | 10.18 | 10.32 | 17.29 | 25.25 | 20.45 | 12.65 | 6.31 |

Where, SH15: Seedling height (15 DAS), SH30: Seedling height (30 DAS), SH45: Seedling height (45 DAS), NT30: Number of tillers (30 DAS), NT45: Number of tillers (45 DAS), NL30: Number of leaves (30 DAS), NL45: Number of leaves (45 DAS), LA15: Leaf area (15 DAS), LA30: Leaf area (30 DAS), LA45: Leaf area (45 DAS), SFW15: Shoot fresh weight (15 DAS), SFW30: Shoot fresh weight (30 DAS), SFW45: Shoot fresh weight (45 DAS), SDW15: shoot dry weight (15 DAS), SDW30: shoot dry weight (30 DAS), SDW45: shoot dry weight (45 DAS)

| **Source** | **DF** | **AGR15** | **AGR30** | **AGR45** | **SLA15** | **SLA30** | **SLA45** | **LAI15** | **LAI30** | **LAI45** |
| --- | --- | --- | --- | --- | --- | --- | --- | --- | --- | --- |
| Block (ignoring Treatments) | 3 | 0.07 ** | 0.09 ** | 0.04 ns | 5088.18 ns | 12531.69 ** | 5593.73 ns | 0.01 ** | 0.35 ** | 10.54 ** |
| Treatment (eliminating Blocks) | 148 | 0.01 * | 0.04 * | 0.09 * | 9649.2 * | 4265.12 ns | 5959.01 * | 0.003 ** | 0.11 ** | 2.14 ** |
| Treatment: Check | 4 | 0.15 ** | 0.29 ** | 0.13 * | 60958.41 ** | 7447.05 * | 6904.04 ns | 0.04 ** | 0.39 ** | 4.92 ** |
| Treatment: Test and Test vs. Check | 144 | 0.01 * | 0.03 * | 0.09 * | 8223.94 * | 4176.73 ns | 5932.76 * | 0.0019 ** | 0.11 ** | 2.06 ** |
| Treatment (ignoring Blocks) | 148 | 0.02 ** | 0.04 * | 0.09 * | 9466.44 * | 4419.96 ns | 5615.73 * | 0.003 ** | 0.12 ** | 2.35 ** |
| Treatment: Test vs. Check | 1 | 0.11 ** | 0.1 * | 1.19 ** | 7242.02 ns | 3172.29 ns | 5307.25 ns | 0.19 ** | 1.45 ** | 44.2 ** |
| Treatment: Test | 143 | 0.01 * | 0.03 * | 0.08 * | 8041.66 ns | 4344.01 ns | 5581.85 * | 0.00052 ns | 0.1 ** | 1.98 ** |
| Block (eliminating Treatments) | 3 | 0.0027 ns | 0.01 ns | 0.09 ns | 14104.19 * | 4892.76 ns | 22528.79 ** | 0.01 ** | 0.0013 ns | 0.1 ns |
| Residuals | 12 | 0.0045 | 0.01 | 0.03 | 3508.37 | 2029.36 | 2233.73 | 0.00042 | 0.01 | 0.11 |
| CV(%)@0.05 |  | 8.08 | 21.1 | 27.04 | 16.79 | 14.11 | 18.93 | 17.13 | 9.21 | 10.18 |

| **Source** | **DF** | **LAR15** | **LAR30** | **LAR45** | **CGR15** | **CGR30** | **CGR45** | **RGR30** | **RGR45** |
| --- | --- | --- | --- | --- | --- | --- | --- | --- | --- |
| Block (ignoring Treatments) | 3 | 331.8 ns | 1949.69 * | 741.2 ns | 0.0012 ** | 0.02 ** | 5 ** | 0.00069 * | 0.002 ** |
| Treatment (eliminating Blocks) | 148 | 3482.8 ns | 3228.59 ** | 2568.54 ** | 0.00022 ns | 0.04 ** | 0.95 ** | 0.0014 ** | 0.00094 ** |
| Treatment: Check | 4 | 19203.61 ** | 51147.71 ** | 16443.83 ** | 0.002 ** | 0.06 ** | 0.69 ** | 0.0042 ** | 0.0012 ** |
| Treatment: Test and Test vs. Check | 144 | 3046.11 ns | 1897.5 ** | 2183.11 * | 0.00017 ns | 0.03 ** | 0.95 ** | 0.0013 ** | 0.00093 ** |
| Treatment (ignoring Blocks) | 148 | 3440.88 ns | 3265.4 ** | 2460.09 ** | 0.00022 ns | 0.04 ** | 1.04 ** | 0.0013 ** | 0.00097 ** |
| Treatment: Test vs. Check | 1 | 190215.1 ** | 214925.93 ** | 109121.38 ** | 0.0014 ** | 0.98 ** | 24.09 ** | 0.07 ** | 0.01 ** |
| Treatment: Test | 143 | 1693.85 ns | 445.89 ns | 1323.06 ns | 0.00016 ns | 0.03 ** | 0.89 ** | 0.00078 ** | 0.00091 ** |
| Block (eliminating Treatments) | 3 | 2399.64 ns | 133.87 ns | 6091.21 ** | 0.0011 ** | 0.0047 ns | 0.11 ** | 0.0026 ** | 0.00037 ns |
| Residuals | 12 | 1573.62 | 352.46 | 689.79 | 9.90E-05 | 0.0034 | 0.02 | 0.00019 | 0.00019 |
| CV(%)@0.05 |  | 21.33 | 10.95 | 19.51 | 20.45 | 15 | 8.28 | 9.55 | 14.1 |

Where, AGR15: Absolute growth rate (15 DAS), AGR30: Absolute growth rate (30 DAS), AGR45: Absolute growth rate (45 DAS), SLA15: Specific leaf area (15 DAS), SLA30: Specific leaf area (30 DAS), SLA45: Specific leaf area (45 DAS), LA15: Leaf area index (15 DAS), LA30: Leaf area index (30 DAS), LA45: Leaf area index (45 DAS), LAR15: Leaf area ratio (15 DAS), LAR30: Leaf area ratio (30 DAS), LAR45: Leaf area ratio (45 DAS), CGR15: crop growth rate (15 DAS), CGR30: crop growth rate (30 DAS), CGR45: crop growth rate (45 DAS), RGR30: Relative growth rate (30 DAS) and RGR45: Relative growth rate (45 DAS).

**Supplementary Table S2. Phenotypic variation for weed competitive associated traits in mapping population and parents**

| **Sl. No.** | **Trait name** | **IR64** | **IRGC15089** | **BC_1_F_2:3_ population** | | | **Skewness** |
| --- | --- | --- | --- | --- | --- | --- | --- |
|  |  |  |  | **Min** | **Max** | **Mean±SD** |  |
| **1** | **SH15** | 13.79 | 13.17 | 9.00 | 16.37 | 12.29±1.50 | 0.07^ns^ |
| **2** | **SH30** | 18.79 | 22.91^*^ | 14.53 | 30.30 | 20.56±2.97 | 0.50^*^ |
| **3** | **SH45** | 20.73 | 29.00 ^*^ | 17.90 | 43.33 | 30.30±4.95 | 0.33^ns^ |
| **4** | **NT30** | 2.31 | 3.63 ^*^ | 1.67 | 8.00 | 3.99±0.94 | 1.01^**^ |
| **5** | **NT45** | 4.57 | 5.23 ^*^ | 3.67 | 21.00 | 9.24±2.58 | 1.07^**^ |
| **6** | **NL15** | 3.63 | 2.97 | 3.00 | 3.00 | 3.00 | - |
| **7** | **NL30** | 7.60 | 10.45 ^*^ | 5.00 | 28.00 | 14.28±3.54 | 0.58^**^ |
| **8** | **NL45** | 14.72 | 18.24 ^*^ | 12.67 | 72.00 | 32.46±8.74 | 0.90^**^ |
| **9** | **LA15** | 6.63 | 13.58 ^*^ | 1.20 | 6.00 | 3.58±1.15 | 5.12^**^ |
| **10** | **LA30** | 15.10 | 42.20 ^*^ | 10.47 | 80.10 | 32.68±10.69 | 0.97^**^ |
| **11** | **LA45** | 46.88 | 105.08 ^*^ | 26.46 | 270.00 | 116.82±46.58 | 0.74^**^ |
| **12** | **SFW15** | 0.12 | 0.20 ^*^ | 0.037 | 0.153 | 0.090±0.02 | 1.53^**^ |
| **13** | **SFW30** | 0.42 | 0.91 ^*^ | 0.39 | 4.23 | 1.04±0.43 | 3.27^**^ |
| **14** | **SFW45** | 1.51 | 2.66 ^*^ | 1.15 | 13.18 | 5.09±2.1 | 0.83^**^ |
| **15** | **SDW15** | 0.026 | 0.036 ^*^ | 0.005 | 0.040 | 0.021±0.1 | 0.25^ns^ |
| **16** | **SDW30** | 0.038 | 0.170 ^*^ | 0.067 | 0.560 | 0.210±0.08 | 1.45^**^ |
| **17** | **SDW45** | 0.140 | 0.497 ^*^ | 0.200 | 2.636 | 0.990±0.46 | 0.87^**^ |
| **18** | **AGR15** | 0.92 | 0.88 | 0.60 | 1.09 | 0.819±0.10 | 0.07^**^ |
| **19** | **AGR30** | 0.33 | 0.65 ^*^ | 0.14 | 1.11 | 0.552±0.18 | 0.56^**^ |
| **20** | **AGR45** | 0.13 | 0.41 ^a^ | 0.07 | 1.64 | 0.649±0.29 | 0.69^**^ |
| **21** | **SLA15** | 231.40 | 618.99 | 162.25 | 843.73 | 350.29±92.30 | 1.88^**^ |
| **22** | **SLA30** | 350.53 | 403.33 ^*^ | 108.54 | 501.33 | 320.84±66.08 | 0.36^ns^ |
| **23** | **SLA45** | 299.13 | 342.49 ^*^ | 105.39 | 688.41 | 251.79±74.61 | 2.90^**^ |
| **24** | **LAI15** | 0.20 | 0.41 ^*^ | 0.04 | 0.18 | 0.11±0.03 | 5.12^**^ |
| **25** | **LAI30** | 0.45 | 1.27 ^*^ | 0.31 | 2.41 | 0.98±0.32 | 0.97^**^ |
| **26** | **LAI45** | 1.41 | 3.16 ^*^ | 0.79 | 8.11 | 3.51±1.40 | 0.74^**^ |
| **27** | **LAR15** | 259.58 | 381.82 ^*^ | 111.60 | 441.33 | 173.27±44.92 | 2.92^**^ |
| **28** | **LAR30** | 393.22 | 248.79 ^*^ | 57.88 | 214.29 | 157.94±29.60 | 2.85^**^ |
| **29** | **LAR45** | 335.56 | 211.26 ^*^ | 50.09 | 361.42 | 124.98±40.48 | 3.54^**^ |
| **30** | **CGR15** | 0.06 | 0.08 ^*^ | 0.01 | 0.09 | 0.05±0.01 | 0.25^ns^ |
| **31** | **CGR30** | 0.03 | 0.30 ^*^ | 0.09 | 1.19 | 0.42±0.17 | 1.42^**^ |
| **32** | **CGR45** | 0.23 | 0.73 ^*^ | 0.07 | 5.34 | 1.73±0.95 | 0.94^**^ |
| **33** | **RGR30** | 0.027 | 0.104 ^*^ | 0.07 | 0.26 | 0.15±0.03 | -0.09^ns^ |
| **34** | **RGR45** | 0.086 | 0.072 ^*^ | 0.01 | 0.17 | 0.10±0.03 | -0.32^ns^ |

Where, * indicates, parents performing significantly each other.

**Supplementary Table S3. Genetic variability estimates for weed competitive associated traits in mapping population**

| **Trait** | **GCV** | **PCV** | **h_bs_** | **GAM** |
| --- | --- | --- | --- | --- |
| SH15 | 10.1 | 12.98 | 60.57 | 16.21 |
| SH30 | 10.9 | 14.13 | 59.46 | 17.34 |
| SH45 | 15.22 | 16.35 | 86.64 | 29.23 |
| NL30 | 22.51 | 24.91 | 81.64 | 41.96 |
| NL45 | 22.87 | 27.29 | 70.24 | 39.54 |
| NT30 | 20.63 | 23.79 | 75.16 | 36.89 |
| NT45 | 23.88 | 28.16 | 71.91 | 41.77 |
| LA15 | 9.11 | 20.65 | 19.45 | 8.29 |
| LA30 | 31.93 | 33.16 | 92.68 | 63.41 |
| LA45 | 39.58 | 40.77 | 94.26 | 79.28 |
| SFW15 | 15.96 | 19.25 | 68.76 | 27.31 |
| SFW30 | 38.51 | 41.93 | 84.34 | 72.96 |
| SFW45 | 33.89 | 41.71 | 66 | 56.8 |
| SDW15 | 16.85 | 26.76 | 39.66 | 21.9 |
| SDW30 | 34.86 | 36.9 | 89.25 | 67.94 |
| SDW45 | 46.57 | 46.95 | 98.4 | 95.3 |
| AGR15 | 10.1 | 12.98 | 60.57 | 16.21 |
| AGR30 | 25.99 | 33.31 | 60.86 | 41.82 |
| AGR45 | 36.34 | 44.72 | 66.07 | 60.94 |
| SLA15 | 19.18 | 25.55 | 56.37 | 29.71 |
| SLA30 | 15.02 | 20.57 | 53.28 | 22.61 |
| SLA45 | 23.03 | 29.74 | 59.98 | 36.8 |
| LAI15 | 9.11 | 20.65 | 19.45 | 8.29 |
| LAI30 | 31.93 | 33.16 | 92.68 | 63.41 |
| LAI45 | 39.58 | 40.77 | 94.26 | 79.28 |
| LAR15 | 6.2 | 23.28 | 7.1 | 3.41 |
| LAR30 | 5.98 | 13.06 | 20.95 | 5.65 |
| LAR45 | 19.72 | 28.5 | 47.86 | 28.14 |
| CGR15 | 16.85 | 26.76 | 39.66 | 21.9 |
| CGR30 | 38.11 | 40.68 | 87.74 | 73.63 |
| CGR45 | 55.28 | 55.82 | 98.06 | 112.92 |
| RGR30 | 16.4 | 18.8 | 76.08 | 29.51 |
| RGR45 | 27.1 | 30.42 | 79.32 | 49.79 |

Where,

GCV: Genotypic co-efficient of variability

PCV: Phenotypic co-efficient of variability

h_bs_: Heritability (broad sense)

GAM: Genetic advance as percent mean

**Supplementary Table S4. Details of SSR markers used for linkage map construction along with their genetic distance (cM) and marker interval (cM)**

| **Chromosome** | **Polymorphic markers** | **Total Distance (cM)** | **Marker Interval (cM)** |
| --- | --- | --- | --- |
| **1** | 10 | 159.46 | 15.95 |
| **2** | 8 | 281.11 | 35.14 |
| **3** | 7 | 80.29 | 11.47 |
| **4** | 3 | 64.52 | 21.51 |
| **5** | 7 | 94.12 | 13.45 |
| **6** | 6 | 62.80 | 10.47 |
| **7** | 7 | 139.56 | 19.94 |
| **8** | 6 | 99.67 | 16.61 |
| **9** | 6 | 112.83 | 18.81 |
| **10** | 8 | 56.39 | 7.04 |
| **11** | 7 | 110.89 | 15.84 |
| **12** | 6 | 161.39 | 26.89 |
| **Total** | 81 | 1423 | - |
| **Average** | **7** | **118.60** | **17.76** |

**Supplementary Table. S5: Chromosome wise distribution of weed competitive QTLs**

| **Sl. No** | **Chromosome** | **No. of QTLs** | **QTLs name** |
| --- | --- | --- | --- |
| 1 | **Chromosome2** | 13 | *qSH30-2.1, qLA15-2.1,qFW45-2.1, qDW15-2.1,qDW30-2.1, qAGR30-2.1, qSLA15-2.1, qSLA45-2.1, qSLA45-2.2, qSLA45-2.3, qLAR15-2.1, qCGR30-2.1, qCGR45-2.1* |
| 2 | **Chromosome3** | 4 | *qSH45-3.1, qAGR45-3.1, qLAR15-3.1, qRGR45-3.1* |
| 3 | **Chromosome4** | 1 | *qFW30-4.1* |
| 4 | **Chromosome5** | 13 | *qNL30-5.1, qNL30-5.2, qNT30-5.1, qNT30-5.2, qNT45-5.1, qNT45-5.2, qFW45-5.1, qSLA15-5.1, qSLA15-5.2, qSLA15-5.3, qLAR15-5.1, qLAR15-5.2, qCGR45-5.1* |
| 5 | **Chromosome6** | 3 | *qFW30-6.1, qSLA15-6.1, qLAR15-6.1* |
| 6 | **Chromosome7** | 7 | *qSH30-7.1, qLA15-7.1, qLA15-7.2, qDW45-7.1, qSLA15-7.1, qLAI15-7.1, qLAI15-7.2* |
| 7 | **Chromosome8** | 4 | *qSLA30-8.1, qLAR15-8.1, qLAR15-8.2, qLAR30-8.1* |
| 8 | **Chromosome9** | 12 | *qNT30-9.1, qLA30-9.1, qLA45-9.1, qDW30-9.1, qDW45-9.1, qSLA15-9.1, qLAI30-9.1, qLAI45-9.1, qLAR15-9.1, qLAR30-9.1, qCGR30-9.1, qCGR45-9.1* |
| 9 | **Chromosome10** | 4 | *qNL45-10.1, qNT45-10.1, qSLA15-10.1, qLAR15-10.1* |
| 10 | **Chromosome11** | 3 | *qDW15-11.1, qLAR15-11.1, qCGR15-11.1* |
| 11 | **Chromosome12** | 8 | *qSH15-12.1, qSH30-12.1, qAGR15-12.1, qSLA45-12.1, qSLA45-12.2, qLAR30-12.1, qLAR45-12.1, qLAR45-12.2* |

| **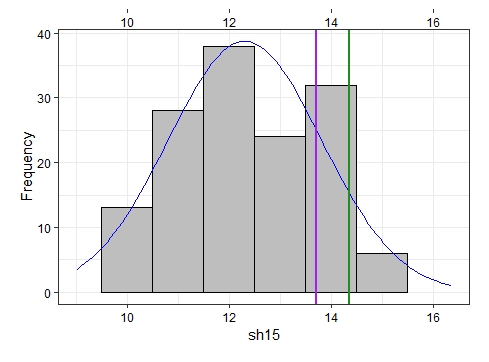** | **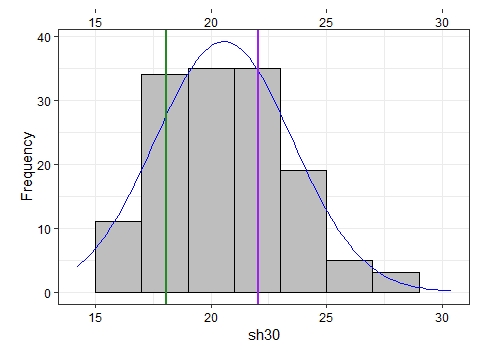** | **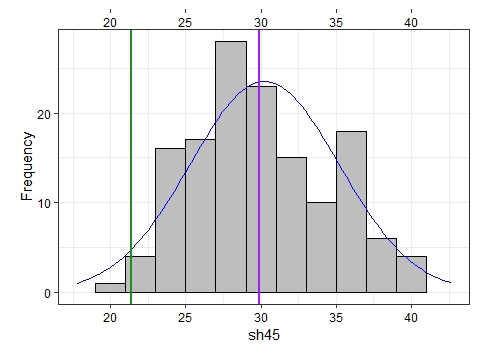** |
| --- | --- | --- |
| **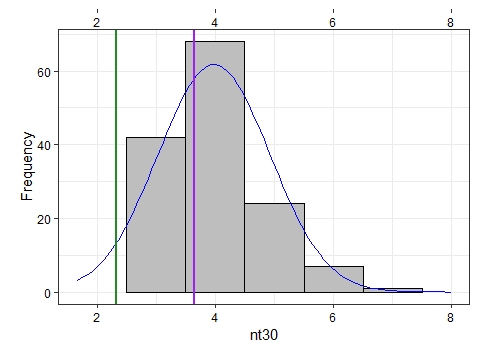** | **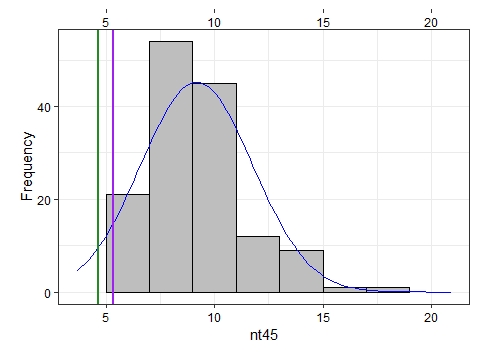** | **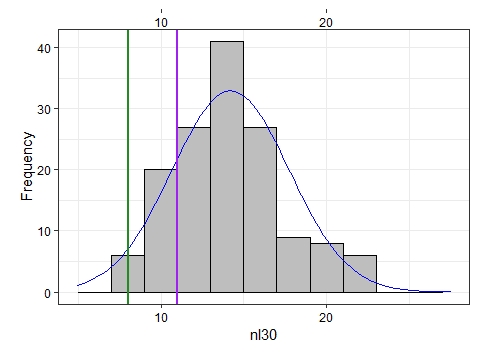** |
| **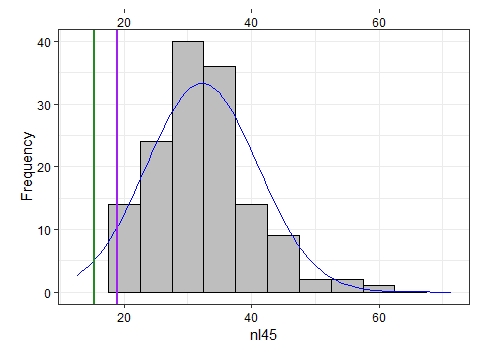** | **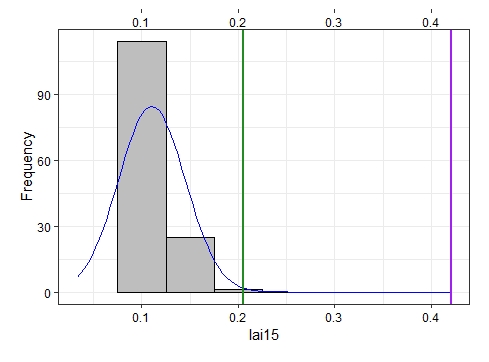** | **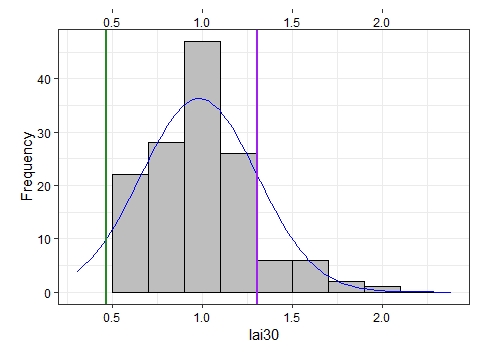** |
| **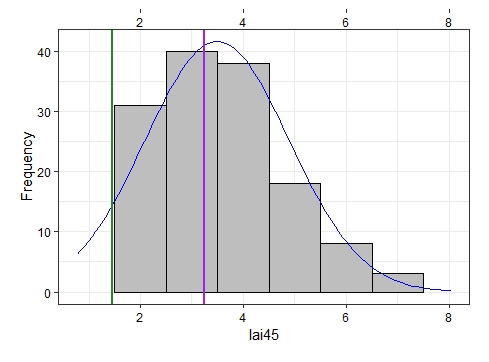** | **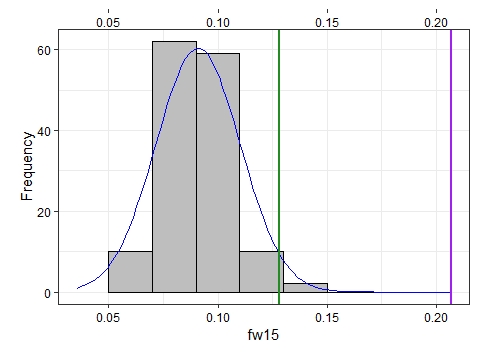** | **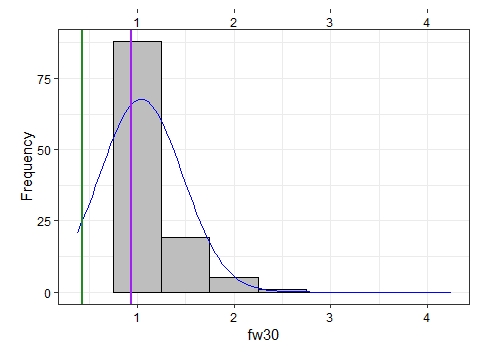** |
| **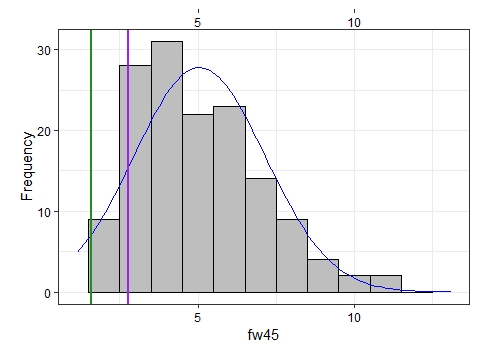** | **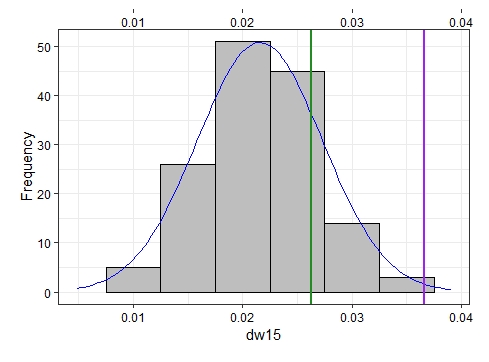** | **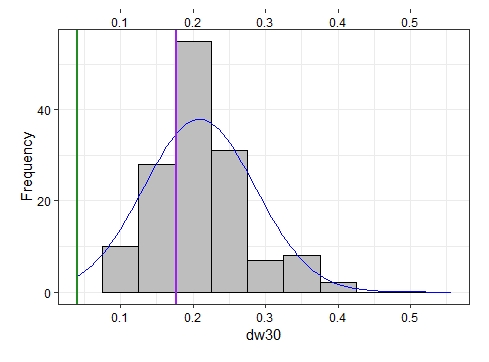** |
| **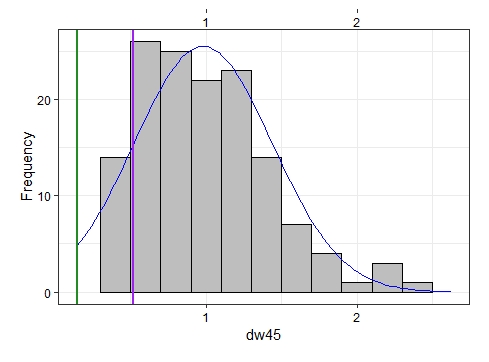** | **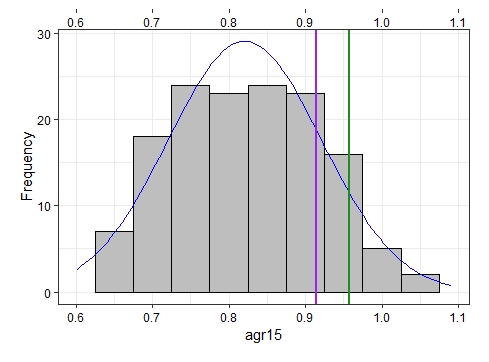** | **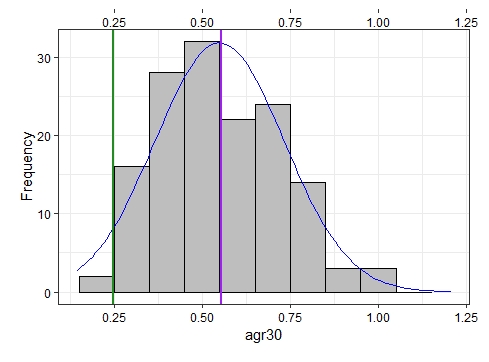** |
| **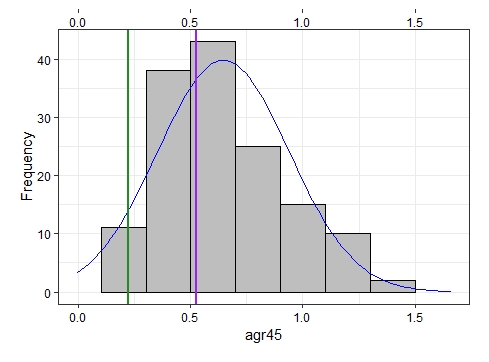** | **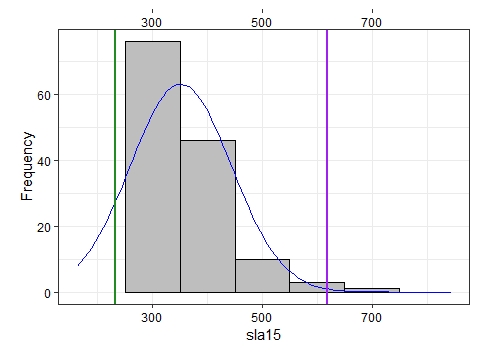** | **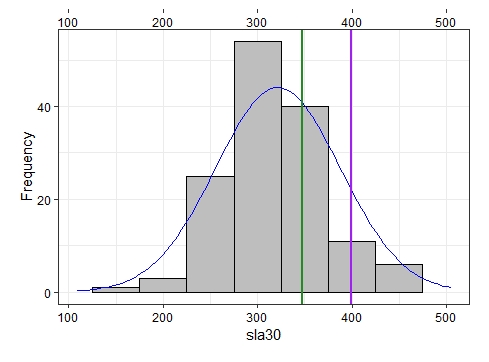** |
| **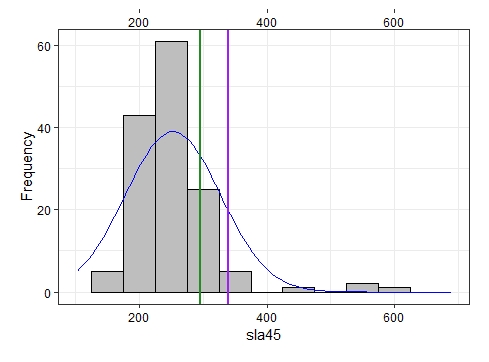** | **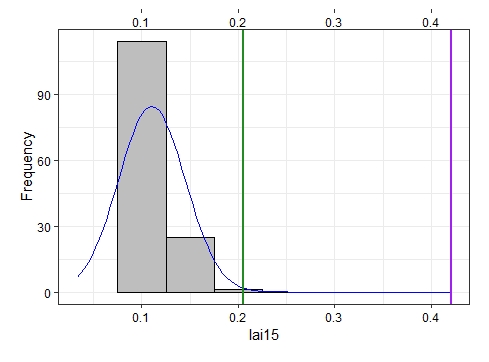** | **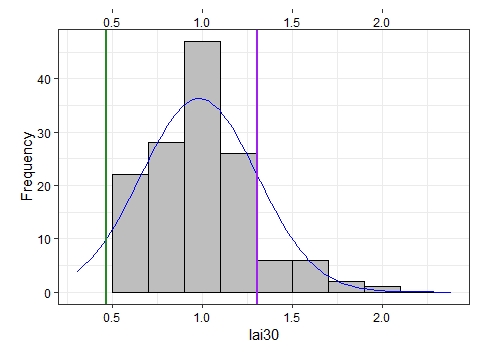** |
| **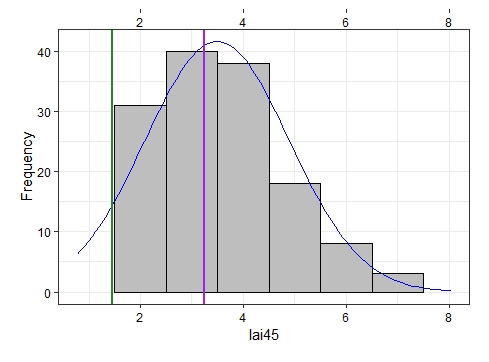** | **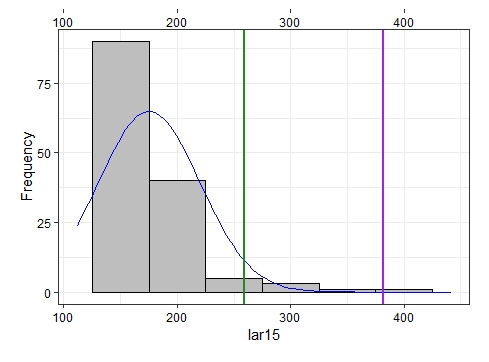** | **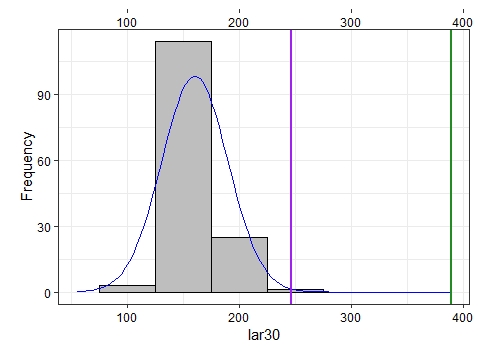** |
| **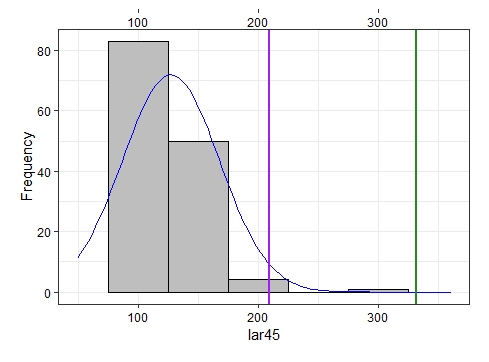** | **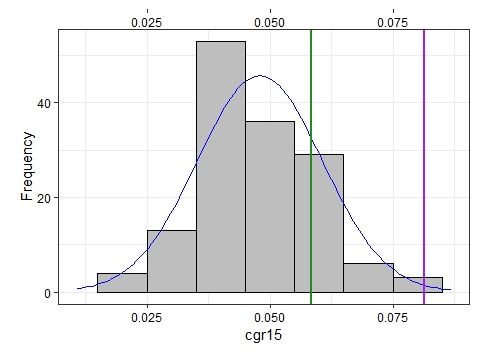** | **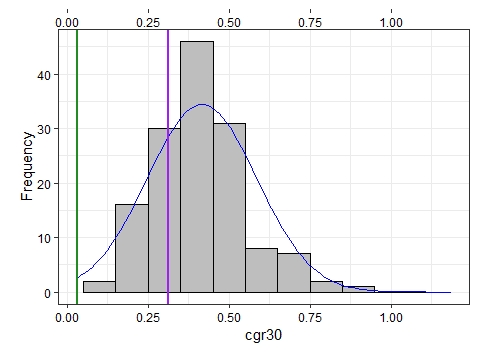** |
| **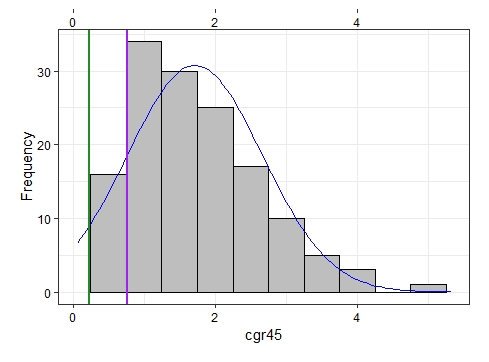** | **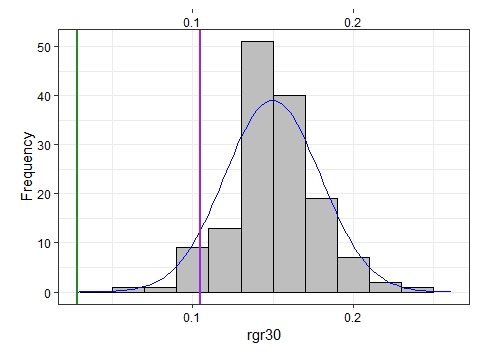** | **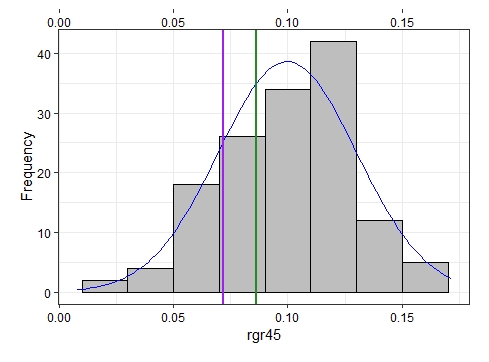** |

**Supplementary Figure. S1: Frequency distribution for weed competitive ability traits in mapping population (Where, green bar: IR64 and Blue bar: *O. glaberrima* parent)**


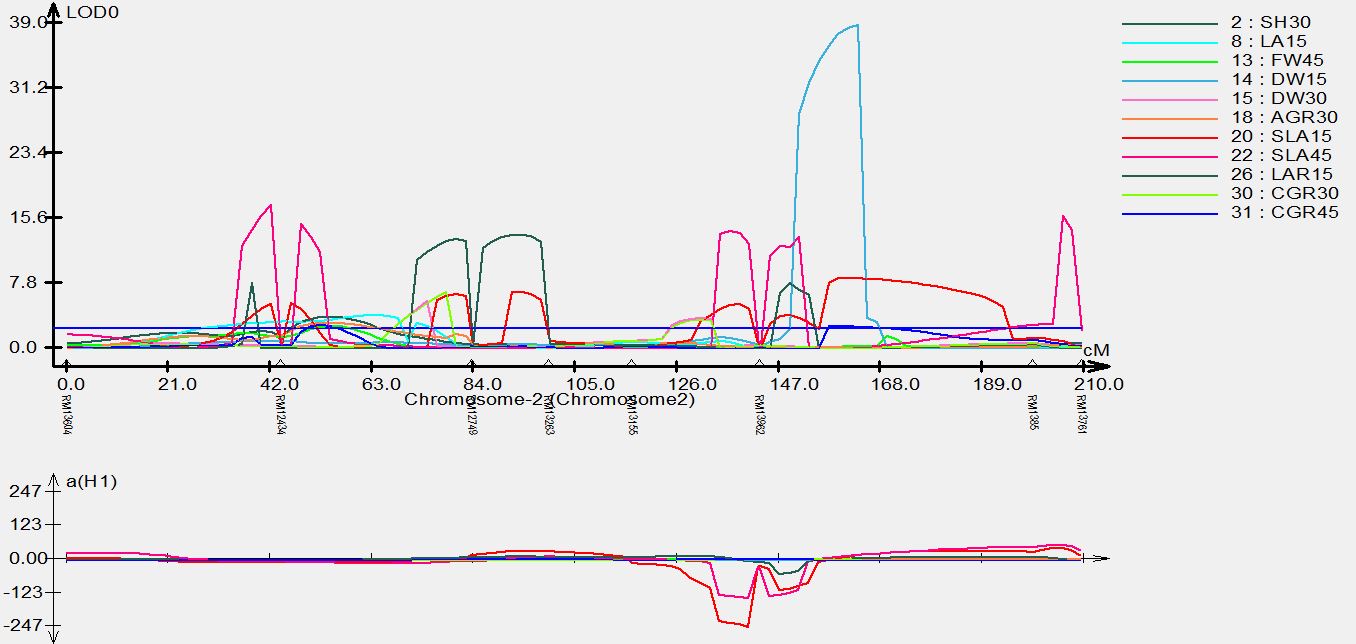

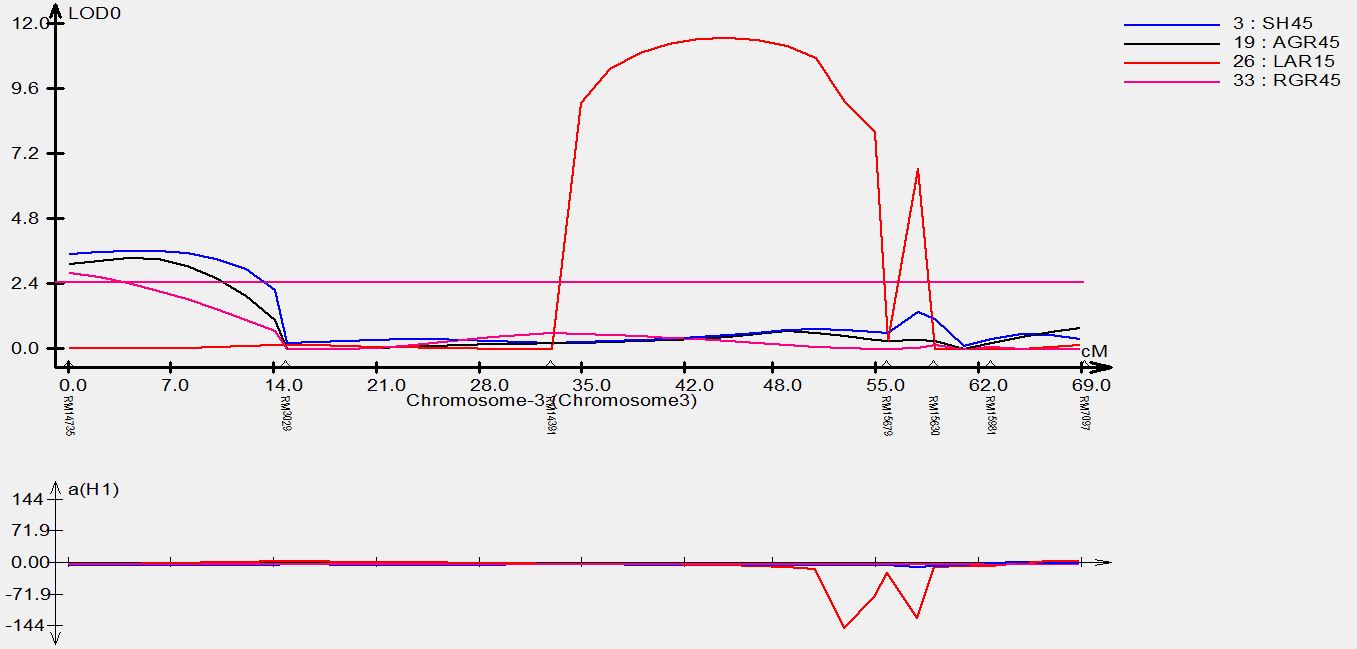

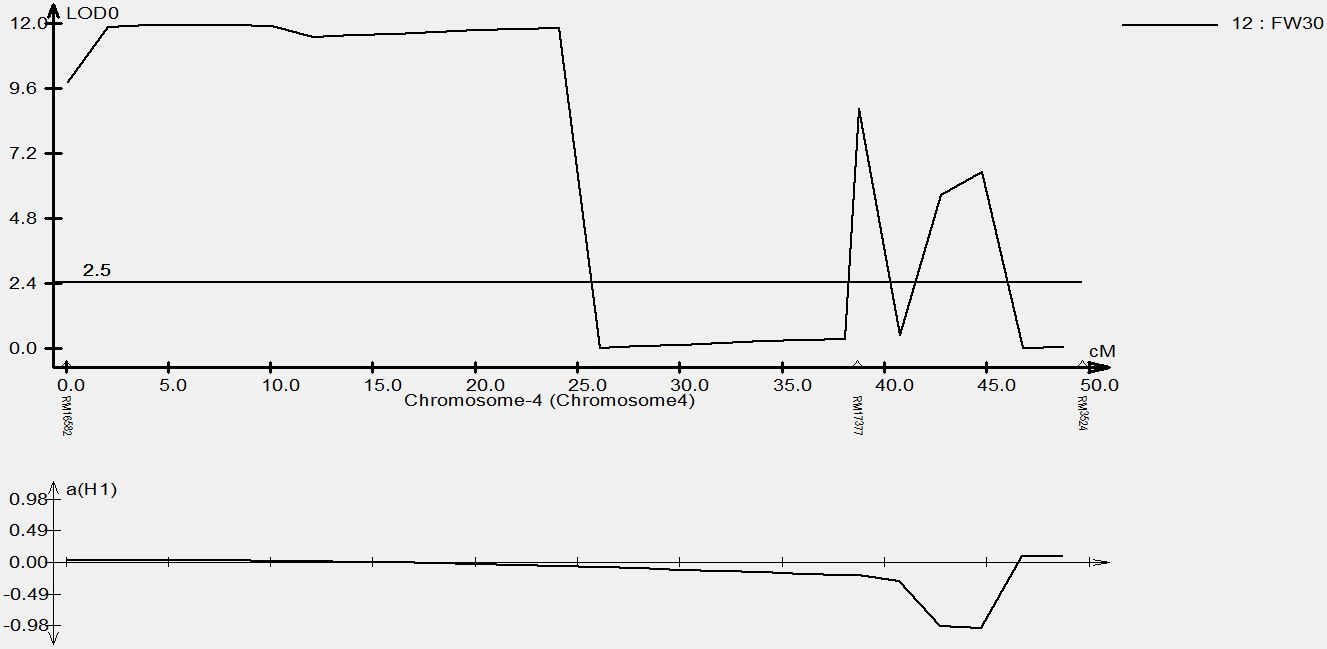

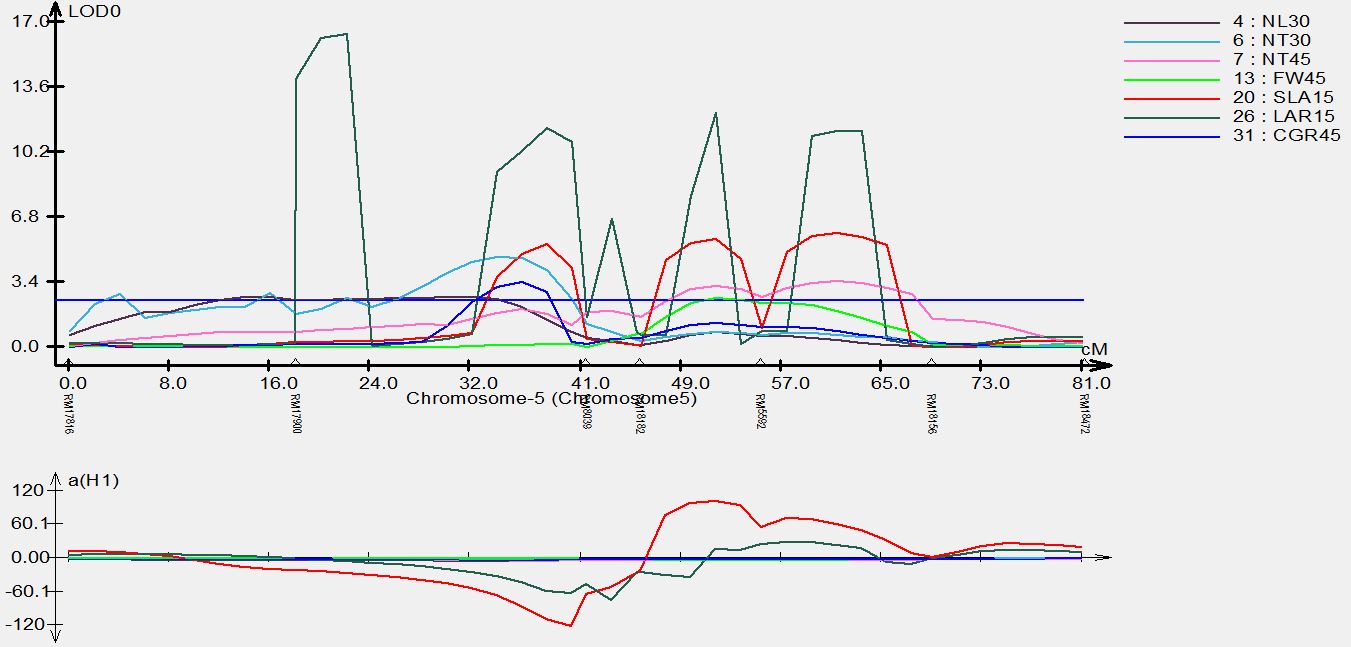

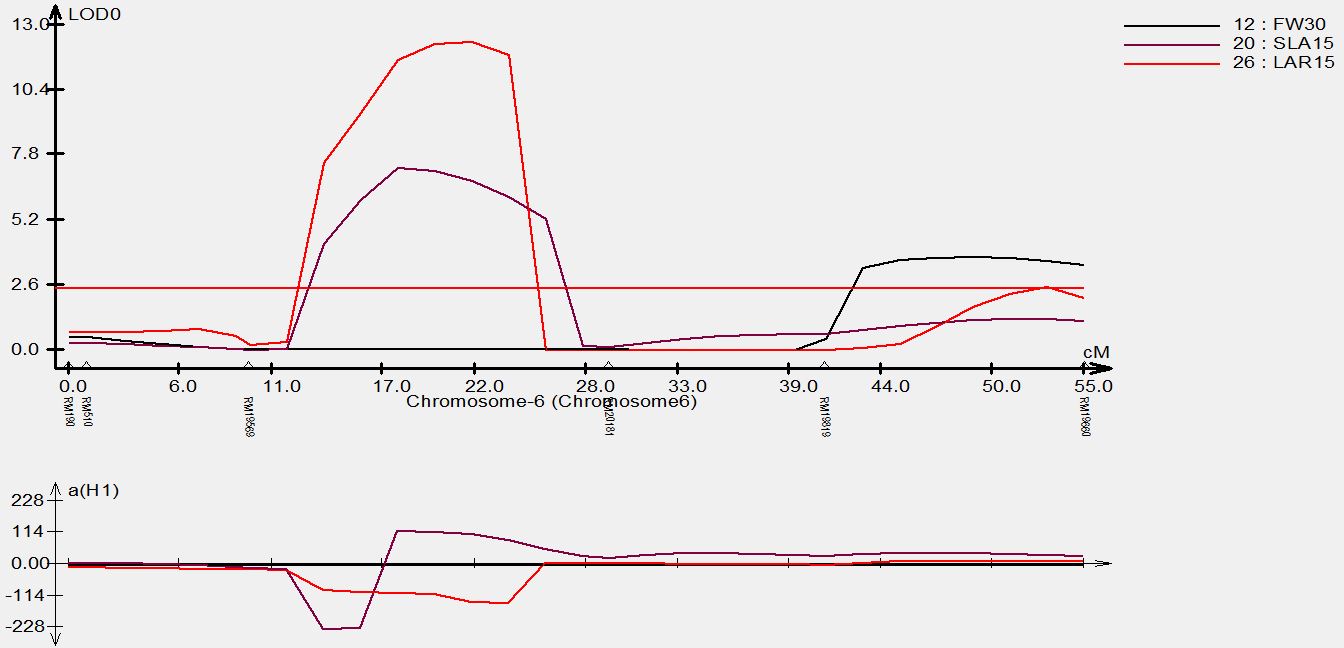

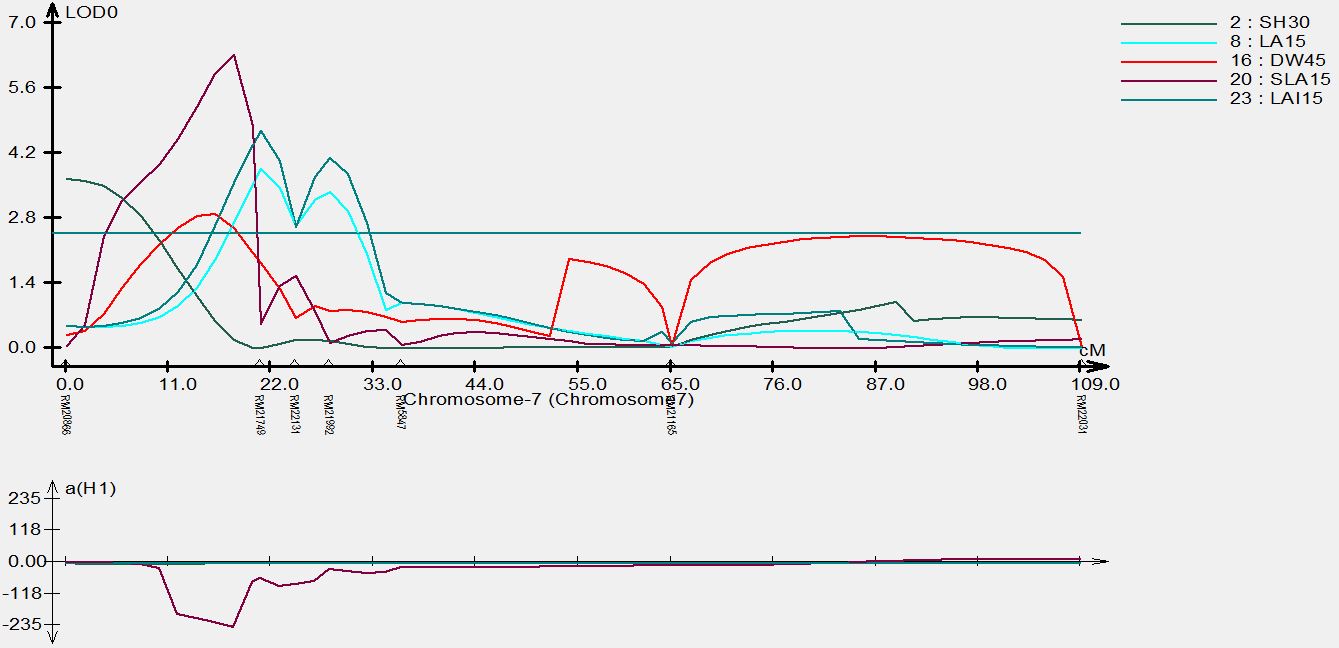

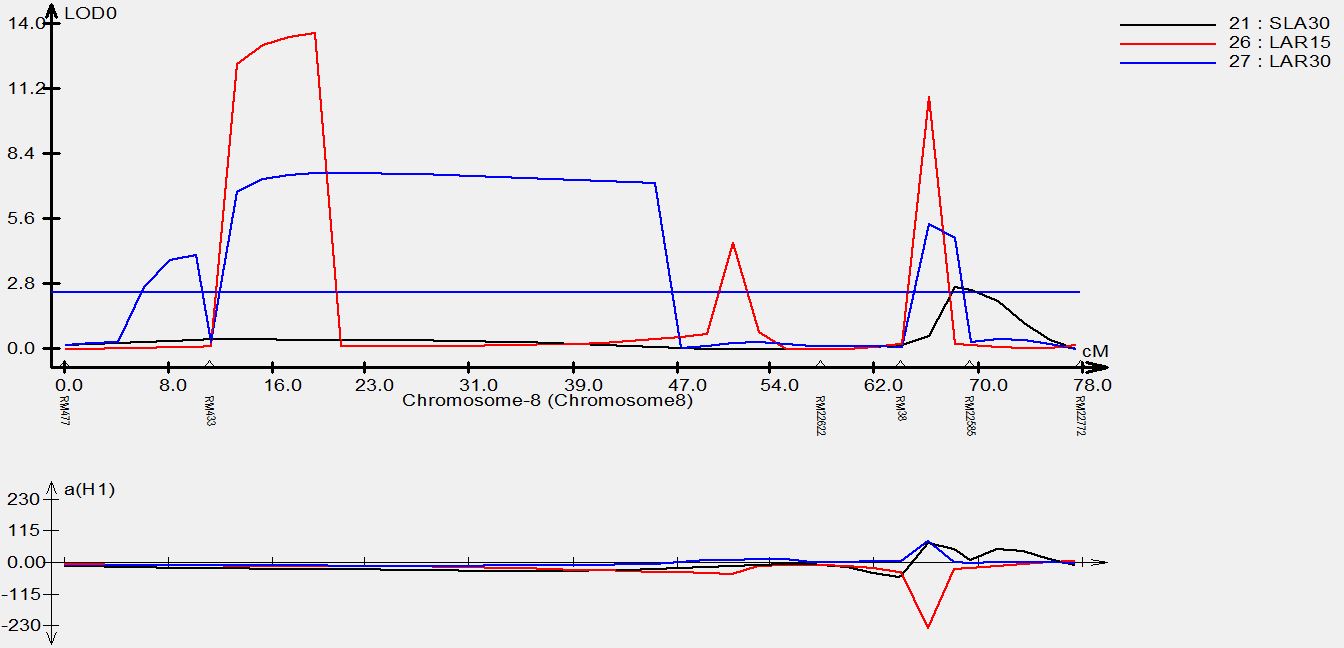

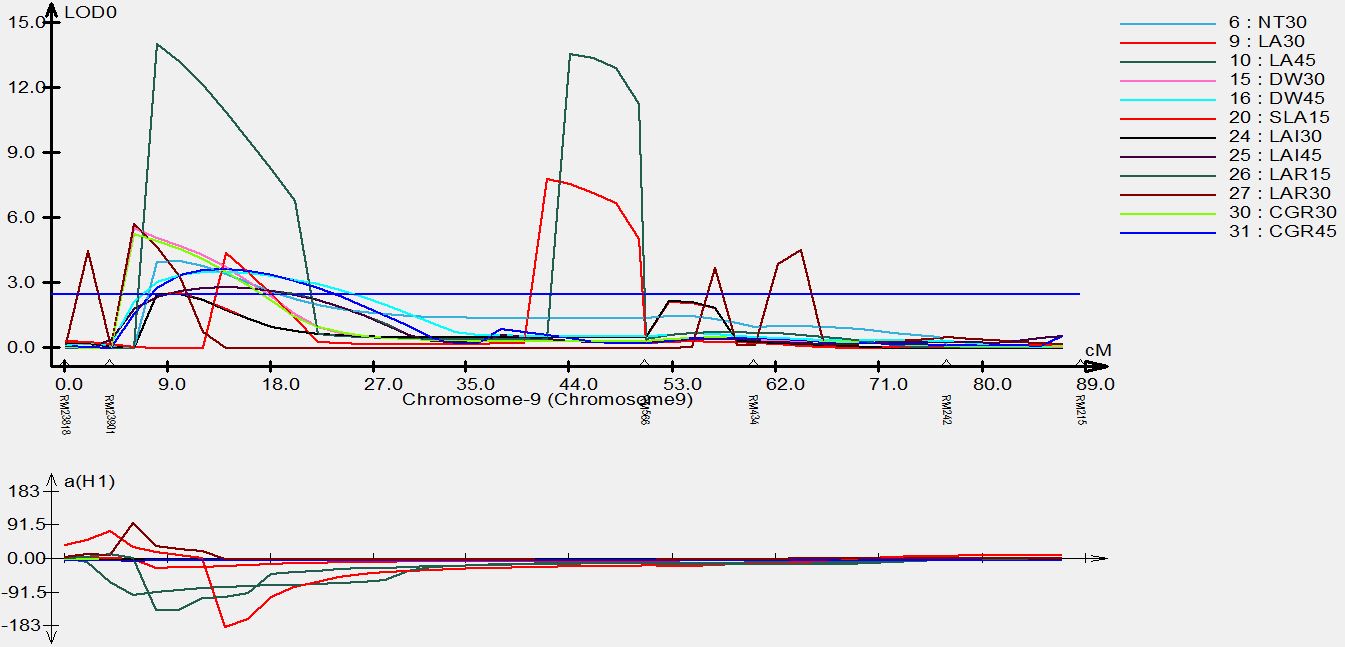

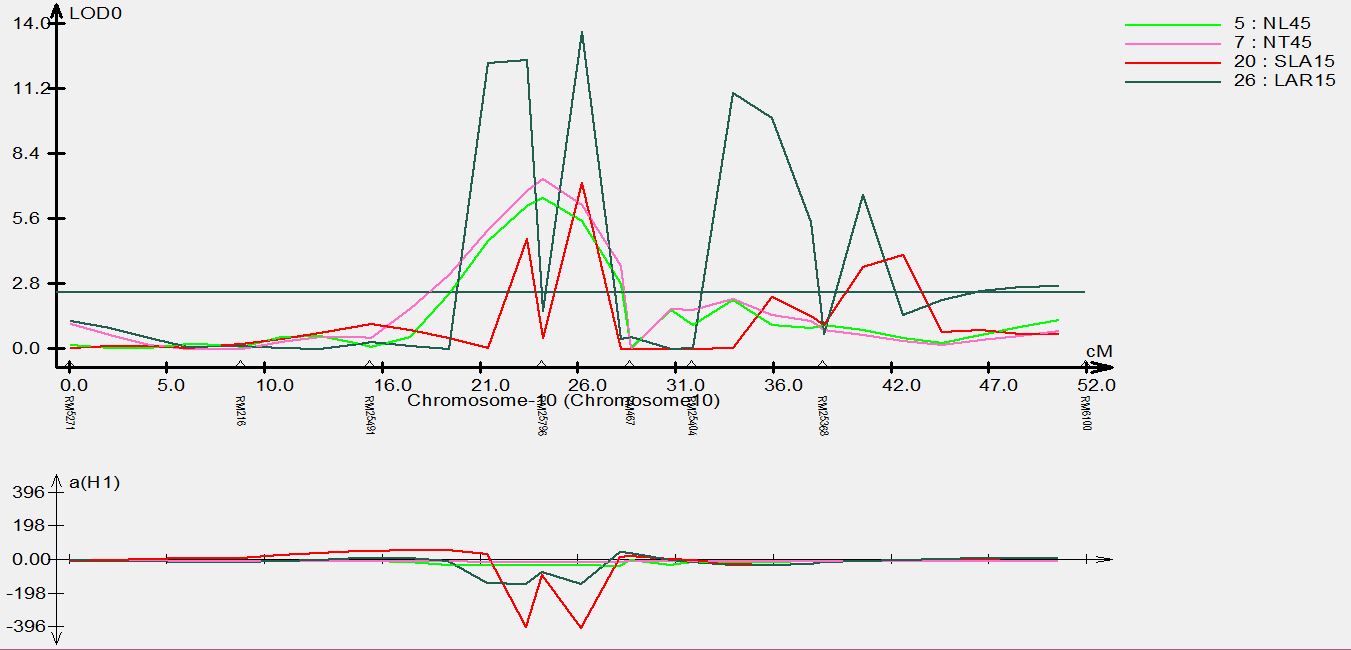


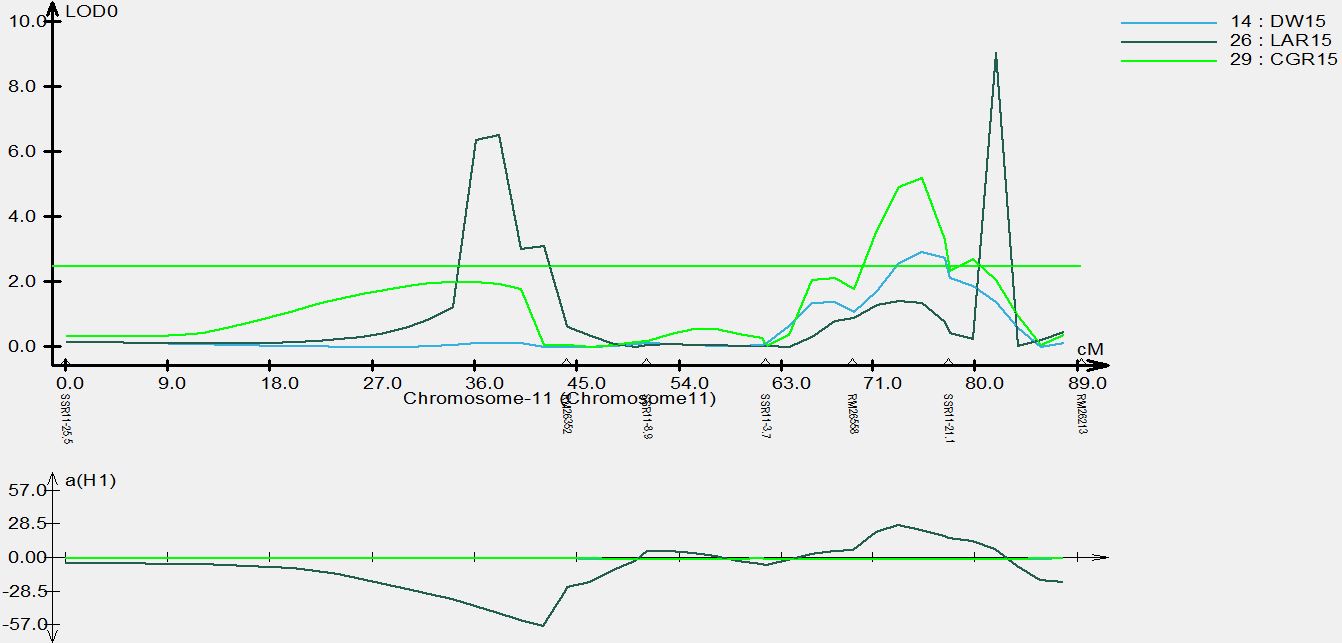

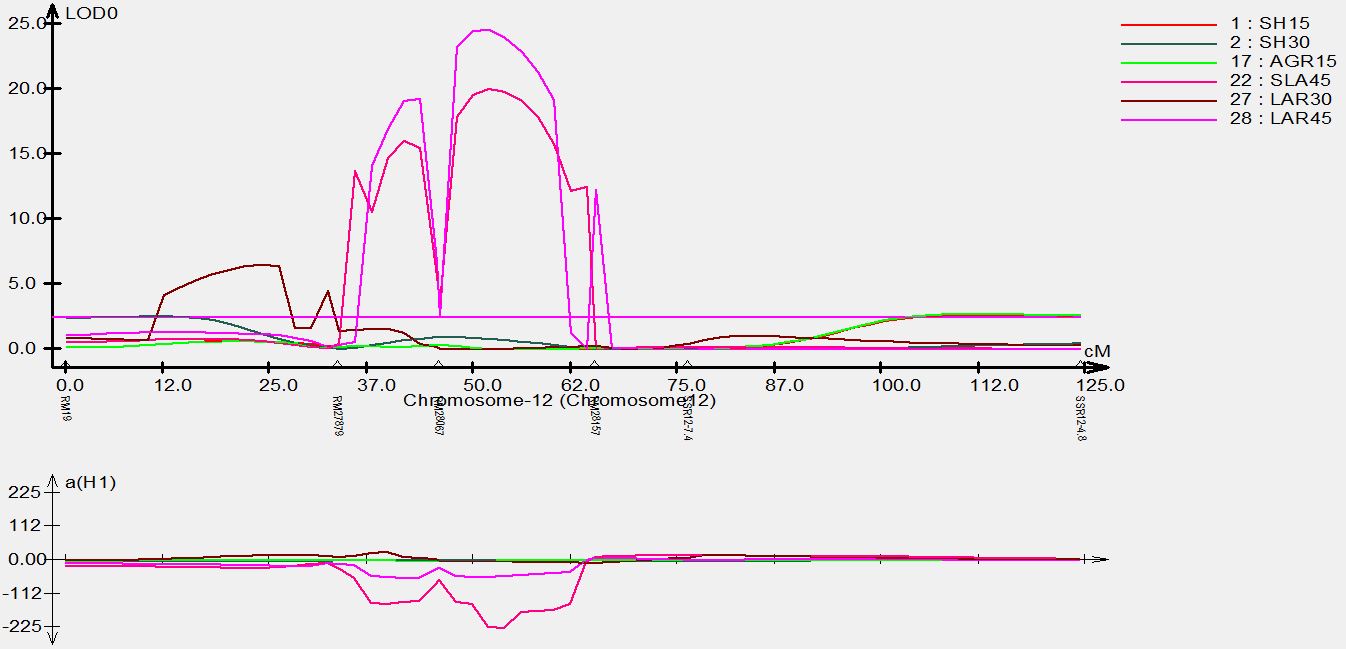


**Supplementary Figure. S2: Graphical representation of QTLs identified for weed competitive ability associated traits ( QTL hotspots were indicated with )**
